# Ultrasound-mediated cavitation enhances the delivery of an EGFR-targeting liposomal formulation designed for chemo-radionuclide therapy

**DOI:** 10.7150/thno.34669

**Published:** 2019-07-28

**Authors:** Eloise Thomas, Jyothi U. Menon, Joshua Owen, Irini Skaripa-Koukelli, Sheena Wallington, Michael Gray, Christophoros Mannaris, Veerle Kersemans, Danny Allen, Paul Kinchesh, Sean Smart, Robert Carlisle, Katherine A. Vallis

**Affiliations:** 1 CRUK/MRC Oxford Institute for Radiation Oncology, Department of Oncology, University of Oxford, Oxford, OX3 7DQ, UK.; 2 Institute of Biomedical Engineering, Department of Engineering Science, University of Oxford, Oxford, OX3 7DQ, UK.

**Keywords:** Ultrasound-Enhanced Delivery, Radionuclide Therapy, Chemotherapy, Breast Cancer, Liposome.

## Abstract

Nanomedicines allow active targeting of cancer for diagnostic and therapeutic applications through incorporation of multiple functional components. Frequently, however, clinical translation is hindered by poor intratumoural delivery and distribution. The application of physical stimuli to promote tumour uptake is a viable route to overcome this limitation. In this study, ultrasound-mediated cavitation of microbubbles was investigated as a mean of enhancing the delivery of a liposome designed for chemo-radionuclide therapy targeted to EGFR overexpressing cancer. **Method:** Liposomes (^111^In-EGF-LP-Dox) were prepared by encapsulation of doxorubicin (Dox) and surface functionalisation with Indium-111 tagged epidermal growth factor. Human breast cancer cell lines with high and low EGFR expression (MDA-MB-468 and MCF7 respectively) were used to study selectivity of liposomal uptake, subcellular localisation of drug payload, cytotoxicity and DNA damage. Liposome extravasation following ultrasound-induced cavitation of microbubbles (SonoVue®) was studied using a tissue-mimicking phantom. *In vivo* stability, pharmacokinetic profile and biodistribution were evaluated following intravenous administration of ^111^In-labelled, EGF-functionalised liposomes to mice bearing subcutaneous MDA-MB-468 xenografts. Finally, the influence of ultrasound-mediated cavitation on the delivery of liposomes into tumours was studied. **Results:** Liposomes were loaded efficiently with Dox, surface decorated with ^111^In-EGF and showed selective uptake in MDA-MB-468 cells compared to MCF7. Following binding to EGFR, Dox was released into the intracellular space and^ 111^In-EGF shuttled to the cell nucleus*.* DNA damage and cell kill were higher in MDA-MB-468 than MCF7 cells. Moreover, Dox and ^111^In were shown to have an additive cytotoxic effect in MDA-MB-468 cells. US-mediated cavitation increased the extravasation of liposomes in an *in vitro* gel phantom model. *In vivo*, the application of ultrasound with microbubbles increased tumour uptake by 66% (p<0.05) despite poor vascularisation of MDA-MB-468 xenografts (as shown by DCE-MRI). **Conclusion:** ^111^In-EGF-LP-Dox designed for concurrent chemo-radionuclide therapy showed specificity for and cytotoxicity towards EGFR-overexpressing cancer cells. Delivery to tumours was enhanced by the use of ultrasound-mediated cavitation indicating that this approach has the potential to deliver cytotoxic levels of therapeutic radionuclide to solid tumours.

## Introduction

**Figure 1:**
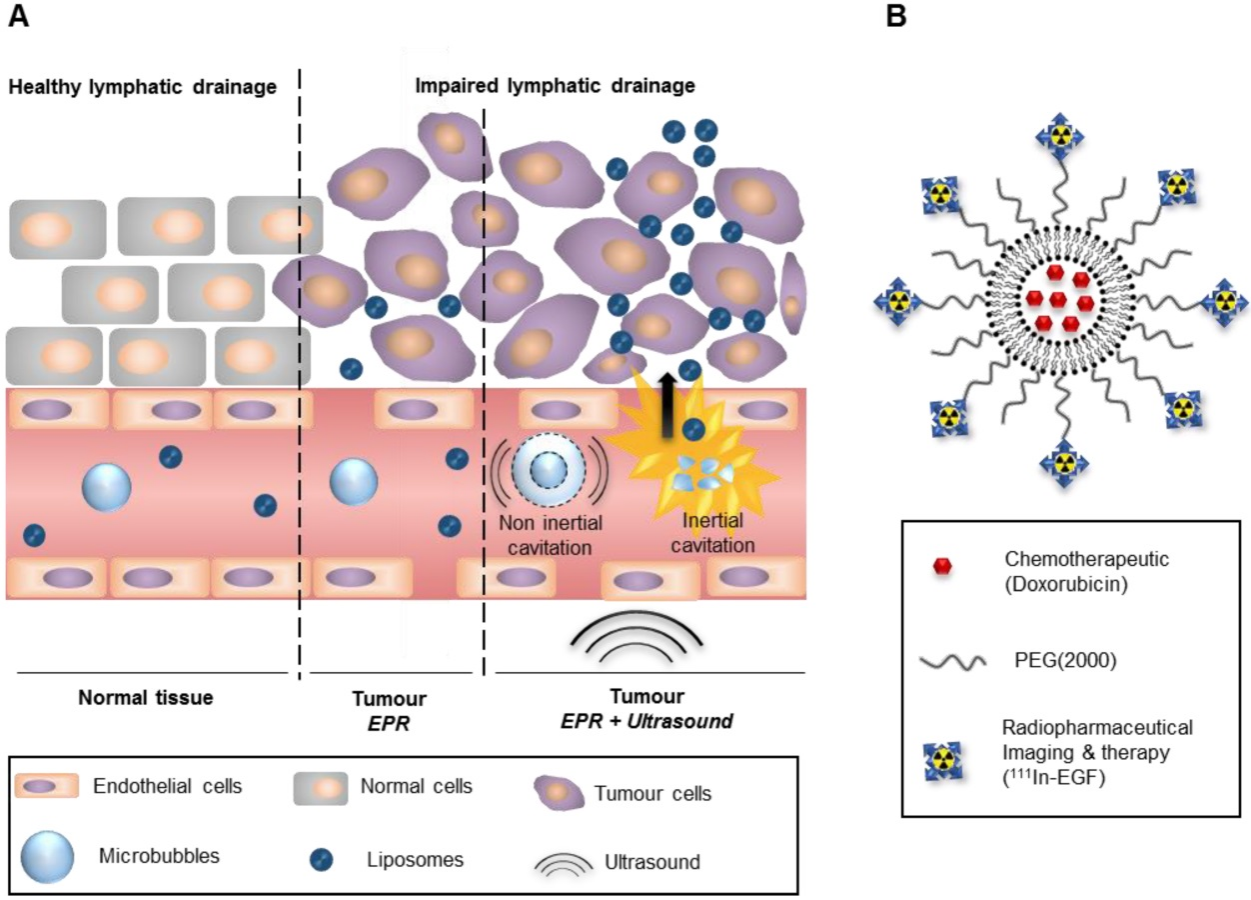
** Combination of US-mediated cavitation and of liposomal formulation for the delivery of chemo-radionuclide to tumours. (A)** Use of ultrasound and microbubbles for cavitation mediated improved delivery. **(B)** Liposomal formulation for a targeted chemo-radionuclide therapy.

Many studies over the last two decades have explored the potential of nanoparticles as drug delivery systems in oncology. These systems have been shown to enhance the efficacy of anticancer drugs and reduce their side effects by improving pharmacokinetics and bioavailability 1-4. This is mainly attributed to the biodistribution of nanoparticles as they are associated with protracted blood circulation and can passively target and accumulate in tumours through the Enhanced Permeability and Retention (EPR) effect (see Figure 1A). The ease with which active targeting, imaging or therapeutic moieties can be added to nanoparticles is another advantage. For example, versatile nanomedicines that carry complementary imaging and therapeutic components are of great interest for personalised medicine and real-time monitoring of therapy 1-4. Combination therapy is also easily achievable through nanomedicine.

Chemoradiotherapy is widely used in cancer medicine as it is associated with reduced loco-regional cancer recurrence and more effective control of metastases compared to single modality treatments 5, 6. However, combined therapy is often associated with significant toxicity 7, 8. A possible solution that retains the benefits of combination treatments but avoids the associated additional toxicity is to deliver both radiation and chemotherapy systemically, for synchronous, spatially-precise targeting of all involved sites. This could be achieved through the design of tumour-seeking nanoparticles that carry both chemotherapy and therapeutic radionuclides.

In this paper, the design (see Figure 1B), synthesis and characterisation of a liposomal formulation for chemo-radionuclide therapy is reported. The core structure is similar to the well-described formulation Doxil®, a liposome encapsulating doxorubicin (Dox) 9, 10. Doxil® dramatically enhances the blood circulation time of its payload and accumulates in tumours where Dox can intercalate into the cellular DNA leading to disruption of topoisomerase-II mediated DNA replication/repair 11. In addition, the liposome designed for the study reported here is surface-functionalised with DTPA-tagged epidermal growth factor (DTPA-EGF). EGF is the natural ligand of the EGF receptor (EGFR) which is overexpressed in several types of cancer including squamous cell head and neck (90-100%), non-small cell lung (75-90%), colorectal (80-85%), breast (20-30%), and cervical (87-100%) cancers and glioma (90-100%) 12. Conjugation of DTPA to EGF allows subsequent radiolabelling with the radiometal Indium-111 (^111^In), which can be used in low amounts for imaging (Single Photon Emission Computed Tomography, SPECT) or in higher amounts for therapy. Indeed, after cell surface binding, ^111^In-tagged EGF shuttles to the cell nucleus where short path-length Auger electrons (< 20 nm in biological media) emitted from ^111^In induce DNA damage 13-17. The liposome designed for this study (hereafter referred to as ^111^In-EGF-LP-Dox) can therefore be used for SPECT imaging and targeted chemo-radiation therapy by combining the topoisomerase inhibition activity of Dox with the direct DNA damage caused by ^111^In; two mechanisms that have been shown to provide synergy 18.

Despite the keen interest in nanoparticles for medicine, recent reviews have highlighted that their accumulation in tumours is limited to an average of 0.7% of the injected dose 19, and that they accumulate mainly in the perivascular space often at the periphery of the tumour 20. These observations are a consequence of the high interstitial fluid pressure, dense extracellular matrix and inefficient and disorganised vessels within tumours 21. To overcome these obstacles and improve the delivery and distribution of anticancer therapeutics, studies have investigated the use of tumour vascular normalisation 22 or the application of physical stimuli including ultrasound (US) 23. As a drug delivery tool US has the advantages that it is widely accessible, non-invasive, relatively low cost and can be precisely targeted to the tumour 20. Delivery of nanoparticles to solid tumours and/or the release of their drug payload can be mediated by the combination of US and gas-filled microbubbles which results in non-inertial cavitation or inertial cavitation of the microbubbles depending on the US parameters applied 24, 25 (see Figure 1A). Non-inertial cavitation consists of the repetitive contraction and expansion of bubbles, in synchronisation with the alternating compressional and rarefactional cycles of the US wave, leading to enhanced local fluid motion and shear stresses on nearby cells. With higher US pressure, the microbubbles violently collapse under the inertia of the surrounding media, generating substantially enhanced convective forces as well as cell membrane perforation and blood vessel permeabilisation 26. Cavitation induced microstreaming can further act as a microscale pump to transport therapeutic agents at millimetre scales. Several studies have shown both in *in vitro* and *in vivo* models that US-induced cavitation can enhance the extravasation of medicinal compounds by increasing vessel permeability and convective forces 27-31. Notably, US-induced cavitation events also produce distinct acoustic emissions that can be recorded providing useful non-invasive feedback during the course of the procedure 20.

We and others have pursued the development of nanosystems for efficient delivery of radiopharmaceuticals 32-35, but to date, their use in conjunction with physical stimuli to promote local deposition of their payload in tumours has been largely ignored with very few reports on this approach 36 and none that have involved the combination with ligand-receptor interactions. In the present study, US-induced cavitation of SonoVue® microbubbles (SV) was used with the intention of enhancing the delivery of an exemplar liposomal formulation, ^111^In-EGF-LP-Dox, for chemo-radionuclide therapy targeted to breast cancer cells. Specifically, ultrasound-induced cavitation was used to increase intratumoural delivery of liposomes by increasing blood vessel permeability and establishing convective flow. Following delivery into the tumour, liposomes are internalised into EGFR-overexpressing cells via the EGF present on their surface ensuring co-delivery of Dox and ^111^In. The current report provides peliminary *in vitro* and *in vivo* evidence that this strategy merits further investigation.

## Methods

### ^111^In-EGF-LP-Dox synthesis and characterisation

1,2-distearoyl-sn-glycero-3-phosphocholine (DSPC), cholesterol, 1,2-distearoyl-sn-glycero-3-phosphoethanolamine-N-[methoxy(polyethylene glycol)-2000] (ammonium salt) (DSPE-PEG(2000) and 1,2-distearoyl-sn-glycero-3-phosphoethanolamine-N-[carboxy(polyethylene glycol)-2000] (sodium salt) (DSPE-PEG(2000)-COOH) (Avanti Polar Lipids, Alabaster, Alabama, USA) were combined in molar ratio 56:39:2.5:2.5 for preparation of liposomes as described previously 11, 37. Liposomes were extruded through a 200 nm membrane at 65°C and doxorubicin (Dox) was loaded using a sulfate gradient with a drug/lipid ratio up to 0.3:1 (w:w) to give LP-Dox. Diethylenetriaminepentaacetic acid (DTPA) was conjugated to recombinant human EGF (EGF, ThermoFisher Scientific, Waltham, Massachusetts, USA) as described previously and DTPA-EGF was attached to the surface of LP-Dox using *N*-hydroxysuccinimide (NHS)-ester crosslinking chemistry 14, 38. A large excess of EDC (1-ethyl-3-(3-dimethylaminopropyl)carbodiimide hydrochloride) and NHS were added to the LP-Dox in 2-(N-morpholino)ethanesulfonic acid buffer (MES) (0.5 M, pH = 5). After incubation for 30 min at room temperature (RT) with shaking, liposomes were purified using a G50 column (GE Healthcare, Buckinghamshire, UK) eluting with Phosphate Buffered Saline (PBS). DTPA-EGF (30 µg/mg of LP) was added immediately to the liposomes and the solution was shaken overnight at 4 °C. DTPA-EGF-LP-Dox (hereafter referred to as EGF-LP-Dox) was separated from free DTPA-EGF via centrifugal filter units (Amicon® 100 kDa, Merck, Darmstadt, Germany) and using a G75 column and elution with sodium citrate buffer (0.1 M, pH = 5.2). Finally, EGF-LP-Dox was radiolabelled by incubation with ^111^In-chloride (up to 300 MBq/mg of liposomes) for 30-60 min at 4 ^o^C.

When Dox was not needed for a specific experiment, it was replaced by NaCl buffer and in some experiments the ratio ^111^In/LP was reduced. When fluorescent liposomes were needed, rhodamine was encapsulated or incorporated into the lipid bilayer. Freshly prepared liposomes were prepared for each experiment. ^111^In-EGF-LP was diluted in saline or PBS for *in vivo* injection. Details of the liposomal characterisation, purity, stability and radiolabelling are given in Supplementary Information (SI).

### *In vitro* studies

EGFR-high MDA-MB-468 cells and EGFR-low MCF7 cells were cultured in Dulbecco's Modified Eagle Medium (DMEM) supplemented with 10% Fetal Bovine Serum (FBS) and 1% penicillin/streptomycin. Cell lines were maintained at 37°C in an atmosphere of 5% CO2 and sub-cultured using trypsin. Cell lines were authenticated by ATCC (Manassas, Virginia, USA), used at passage numbers 30 or lower and checked to be mycoplasma-free on a monthly basis.

### Western Blot analysis

Western blot analysis was performed as described previously 32. Actin was probed with Anti-beta Actin antibody (ab8227, Abcam, Cambridge, UK) diluted at 1:1000 in 0.5 % powder milk and visualised using Goat anti-Rabbit IgG secondary antibody (65-6120, Invitrogen, Waltham, USA) diluted at 1:2000. EGFR was probed with the EGFR (A-10) antibody (sc-373746, Santa Cruz Biotechnology, Dallas, USA) diluted at 1:200 in 0.5 % powder milk and was visualised using the Rabbit anti-Mouse IgG secondary antibody (61-6520, Invitrogen, Waltham, USA) diluted at 1:2000 in 0.5 % powder milk.

### Competitive binding assay

MDA-MB-468 cells were seeded in 24 wells plates (20 x 10^4^ cells per well) and left to attach overnight. They were then incubated in PBS with cold EGF and ^111^In-EGF (0.3 µg, 90 kBq) or ^111^In-EGF-LP (10 µg of LP equivalent to 0.3 µg of ^111^In-EGF, 90 kBq). After 2 h at 4 °C, cells were washed twice with PBS and lysed with RIPA buffer. The amount of radioactivity in the cell lysates was determined using an automated Wizard gamma counter (Perkin Elmer, Waltham, MA, USA).

### Cellular uptake of ^111^In-EGF-LP

MDA-MB-468 and MCF7 cells (1.6 x 10^4^ cells per well in 48 wells plates) were exposed to ^111^In-EGF-LP (2.5 to 60 µg/mL, 0.3 to 7.3 MBq/mL) with or without co-incubation with cold EGF (115 µg/mL). After 2 h at 37 °C, cells were washed, lysed and the amount of radioactivity in the cell lysates was determined as above. Results were normalised to the number of cells.

### Subcellular localization of ^111^In-EGF-LP

The radioactivity associated with different cellular compartments was determined using Nuclei EZ Prep Nuclei Isolation Kit (Sigma-Aldrich, Dorset, UK). Cells were seeded in 12 wells plates at a seeding density of 5 x 10^4^ cells/well and allowed to attach overnight. The cells were treated with 5 µg/mL (0.6 MBq/mL) of ^111^In-EGF-LP for 2 h at 37°C and then washed with PBS. The membrane-bound fraction was collected using an acid wash (PBS, pH 2.5) followed by a wash with PBS 39. Nuclei EZ buffer was added to the wells and cells were scraped and centrifuged to obtain the cytoplasmic fraction (supernatant). The pellet containing cell nuclei was washed 3 times by re-suspension in PBS and centrifugation. The amount of radioactivity in the fractions was measured using an automated Wizard gamma counter.

### Cellular uptake of fluorescently labelled formulations

Rhodamine-containing EGF-LP (0.1 mg/mL) were added to cells cultured in a Lab-Tek II 8-well glass slide (5 x 10^4^ cells/well) for 24 h. Cells were then fixed in 4% paraformaldehyde. Cell nuclei were stained with DAPI-containing Vectashield mounting medium (Vector Laboratories, Burlingame, CA) and cells were visualised using a Zeiss LSM 780 confocal microscope (Zeiss, Germany).

### Clonogenic assay

Cells were seeded in 6-well plates at seeding densities of 300 and 1000 cells/well for MDA-MB-468 and MCF7 cell lines respectively, and incubated overnight. The following day, the cells were treated for 24 h with LP-Dox, EGF-LP-Dox or ^111^In-EGF-LP-Dox (0 to 15 µg/mL of liposomes). Following treatment, the cells were washed with PBS and medium containing 20% FBS was added. Cells were incubated at 37^o^C and 5% CO_2_. After 14 days, colonies were fixed and stained in 1% methylene blue in methanol. Colonies were counted using a GelCount automated colony counter (Oxford Optronix, Abingdon, UK).

### γH2AX immunostaining

Cells were grown on Lab-Tek II 8-well glass slides (5 x 10^4^ cells/well) for 24 h and then exposed to ^111^In-EGF-LP-Dox, ^111^In-EGF-LP or EGF-LP-Dox for 24 h. Cells were then washed and fixed with 4% paraformaldehyde, permeabilised using 1% Triton X-100, blocked for 1 h using 2% BSA in PBS, and immunostained for γH2AX using an anti-γH2AX (Ser139) primary antibody (EMD Millipore, Hertfordshire, UK) and Alexa fluor 488 (AF488)-labelled secondary antibody. Cell nuclei were stained using DAPI and γH2AX foci were observed using a Zeiss LSM 780 confocal microscope. The number of γH2AX foci per cell was quantified using ImageJ software.

### Blood vessel phantom

The demonstration of acoustically triggered extravasation of liposomes in a blood vessel phantom was performed as previously described for fluorescent nano-beads 40. In brief, a tissue-mimicking flow phantom was prepared from degassed hydrogel composed of 1.0% (w/v) low melting point ultrapure agarose gel (Invitrogen, Carlsbad, CA, USA). A solution of SV (0.25 mg/mL) and rhodamine tagged EGF-LP (EGF-LP-Rh, 60µg/mL) was introduced through a 1 mm channel embedded in the agarose phantom at a flow rate of 0.2 mL/min. A focused ultrasound (FUS) transducer (H102, Sonic Concepts, Bothell, WA, USA) of fundamental frequency 1.1 MHz was used to excite the SV at 2.0 MPa peak negative pressure, 3,000 cycles, 3.3 Hz pulse repetition frequency. A 10 MHz passive cavitation detection (PCD) transducer (V320 Panametrics, Olympus, Waltham, USA) was used to record any acoustic emissions emanating from the FUS-exposed portion of the channel. After excitation, the channels were excised and imaged using an inverted microscope (Eclipse Ti, Nikon Inc, USA). Side and top-view TRITC (Tetramethylrhodamine)-fluorescent images (excitation: 545 nm, emission: 620 nm) were acquired around each exposure location.

### Tumour model

All *in vivo* procedures were conducted in accordance with the Animals Scientific Procedures Act of 1986 (UK) (Project License Numbers 30/3115 and P13B66CD9 issued by the Home Office) and protocols approved by the Committee on the Ethics of Animal Experiments of the University of Oxford.

Female athymic nude mice (6-8 weeks old, average weight of 25 g) were purchased from Charles River. To obtain MDA-MB-468 xenografts, 5x10^6^ cells were mixed 1:1 with Matrigel (final volume of 150 µL) and injected subcutaneously (s.c.) in the right flank. Mice were monitored twice weekly for the appearance of tumours which were measured using callipers. Mice were entered into study approximatively 5 weeks after inoculation when tumour volumes reached 50-100 mm^3^.

### *In vivo* biodistribution

Animals (n = 4) were anaesthetised, cannulated via the lateral tail vein and received ^111^In-EGF-LP (5-8 MBq, 120 µg of lipids) intravenously (i.v.) followed by 50 µL of saline to flush the cannula. At 2.5 and 48 h, mice were euthanized, xenografts and organs were harvested, weighed and the amount of radioactivity counted using an automated Wizard gamma counter. The tumours were frozen and sectioned. Sections were exposed to a phosphor screen for two days and read using a Cyclone® Plus (Perkin Elmer, Waltham, MA, USA).

### Pharmacokinetic studies

Blood clearance was evaluated using SPECT imaging. Animals (n=3) were anaesthetised and placed into a bespoke cradle. ^111^In-EGF-LP (120 µg, 8 MBq) was injected i.v. into the tail vein and mice then imaged for approximately100 min acquiring 200 frames of 30 s focussed on the heart. At the end of the SPECT session, a CT scan was performed and animals euthanized. All images were reconstructed using MILabs reconstruction software v3.24 and analysed using PMOD v.3.37 (PMOD Technologies, Zurich, Switzerland). More details are given in the SI.

To investigate the effect of phagocytosis of liposomes by macrophages on blood clearance and pharmacokinetics, mice received clodronate liposomes (Liposoma, Amsterdam, The Netherlands) by i.v. injection (0.1 mL per 10 g of animal weight). In liposomal form, clodronate is taken up by macrophages in the liver (Kupffer cells), spleen and bone marrow causing apoptosis. It has been shown that clodronate-mediated macrophage depletion is complete 24 h after i.v. injection of clodronate liposomes 41, 42. Therefore, mice (n = 3) received an i.v. injection of clodronate liposomes and the pharmacokinetic profile of the liposomes was studied 24 h post injection (p.i.) using SPECT imaging as described previously.

### DCE-MRI

MRI was performed using a 7.0 T 210 mm horizontal bore VNMRS preclinical imaging system equipped with 120 mm bore gradient insert (Varian Inc, CA) and a 32 mm ID quadrature birdcage coil (Rapid Biomedical GmbH, Germany). DCE-MRI was performed using a respiratory gated 3D spoiled gradient echo scan with 30 μL of a Gd-contrast agent (Omniscan, GE Healthcare) infused via a tail vein cannula over 5 s (see SI for more details) 43.

### Contrast-enhanced imaging

Contrast enhanced US was performed using a Vevo3100 scanner (FUJIFILM Visualsonics, Joop Geesinkweg 140, 1114 AB Amsterdam, Netherlandsusing) with a MX250 probe (Centre Transmit Frequency: 20 MHz, Axial Resolution: 75 μm). SV (50 µL) was administered i.v. to anaesthetised mice while imaging was acquired in Non-Linear Contrast mode. A second injection of SV was administered 10 min later and high amplitude US bursts were applied every 10 s in order to acquire destruction-replenishment curves and study the perfusion kinetics (see SI). VevoLab software was used to trace regions of interest within the tumour and quantify contrast intensity as a function of time.

### US-mediated delivery *in vivo*

Mice were placed on an acoustically transparent mylar bed above a water bath thermostatically controlled at 37 ± 1°C. A 64 mm diameter, 1.1 MHz centre frequency FUS transducer (H102, Sonic Concepts) was positioned in the water directly underneath the animal. The centre of the source housed a 13 mm diameter single element transducer (Panametrics V320-SU-F 1.75PTF) that was used as a PCD with best sensitivity in the 4-10 MHz frequency range. Receiver signals were high-pass filtered (F5081-2P0, Allen Avionics, Mineola, NY, USA), pre-amplified (SR445A, SRS, Sunnyvale, CA, USA), digitized (Handyscope HS3, TiePie Engineering, Netherlands) and streamed to a laptop computer disk. For analysis of cavitation activity, PCD time series data sets were processed in MATLAB (Mathworks, Natick, MA, USA) using Welch's method for power spectrum calculation, implemented with temporal windows of 80 μs duration (2.5% of typical source drive pulse length) with 50% window overlap. Prior to all testing, the nested source and PCD transducers were aligned to a reference mark on the Mylar bed (see SI). Before each treatment, US gel (Aquasonic 100, Parker Labs, Fairfield, NJ, USA, centrifuged to minimize entrapped gas) was applied to the mylar mark where the tumour was then placed. Mice (3 per group) were cannulated under anaesthesia and treated in two groups. One group received ^111^In-EGF-LP (120 µg, 5 MBq) i.v followed by 50µL of saline to flush the canula, and the another received ^111^In-EGF-LP (120 µg, 5 MBq) followed by two boluses of SV microbubbles (50 µL at 10 mg/mL for each bolus) administered 45 s apart as well as tumour targeted US (1.1 MHz. 2.0 MPa peak negative pressure, 3000 cycles, 1.2 s pulse repetition period, 2.5 min total exposure). To maximise the portion of the tumour exposed to US the bed of the Murine Ultrasonic Therapy Apparatus (MUTA) was moved along the length of the tumour during treatment. The incident US pressure field had full-width half-maximum amplitude dimensions of 1.6 mm laterally (spot diameter *d*) and 13.0 mm axially. Given the tumour sizes in this study (typically 2.5 mm in depth and 7 mm in diameter), the tumour depth was fully exposed. The coverage (CV) could be approximated in terms of maximum projected surface area (S_tumour_): CV = 100*(*S_spot_ + Ld*)/ S_tumour_, where *S_spot_* = *πd^2^/4* and *L* is the distance scanned along the length of the tumour during treatment. For typical tumour projected areas of S_tumour_ = 40 mm^2^ and scan distances of L = 7 mm, CV was approximately 33 % during the 2.5 min US exposure. Mice were euthanized 10 min after the liposome injection and organs removed for analysis as described in the previous *in vivo* biodistribution section.

## Results and discussion

### ^111^In-EGF-LP-Dox are designed for high drug loading and stability towards US-induced cavitation of SV

^111^In-EGF-LP-Dox was synthesised in three steps: encapsulation of Dox, surface functionalisation with DTPA-EGF and ^111^In-labelling to give a high radiolabelling yield (>90%, see Methods and SI, Figure S1). The addition of radioisotope as the last step of the synthesis is of interest for *in vivo* studies and clinical translation as it minimises loss of therapeutic efficacy due to decay of the isotope during protracted manufacture. This is a clear advantage in comparison to some previously published nanoparticle-radionuclide constructs where the time required for formulation exceeds the half-life of the radionuclide 44. Physico-chemical characterisation of liposomal formulations are presented in the Figure S1. Briefly, EGF-LP have a hydrodynamic diameter of 140 nm and zeta-potential of -30 mV at pH = 7 consistent with negatively charged DTPA-EGF at neutral pH 45. Up to 300 µg of Dox, 30 µg of EGF and 300 MBq could be loaded per mg of liposome which is high enough for therapeutic purposes for both Dox and ^111^In-EGF 10, 15. Results are in line with published data regarding the loading of Dox and its release 11, 37 as well as the amount of MBq/µg of EGF for the non-grafted peptide 14. Therefore ^111^In-labelling of DTPA-EGF is not adversely affected by its attachment to liposomes. As shown in Figure S1A, the size of the liposome formulation did not change following addition of ^111^In.

^111^In-EGF-LP-Dox was shown to be stable under exposure to US in the presence of SV (see Figure S1). This is in agreement with the strategy being used here which relies on the use of SV cavitation to increase the delivery of the intact liposome into the tumour by increasing blood vessel permeability and flow convection. This stability is desirable as it ensures optimal co-localisation of ^111^In and Dox. In this respect, the approach used here differs from previous studies looking at US-triggered release of drugs from liposomes 46.

### ^111^In-EGF-LP-Dox is taken up selectively in EGFR overexpressing cells

**Figure 2:**
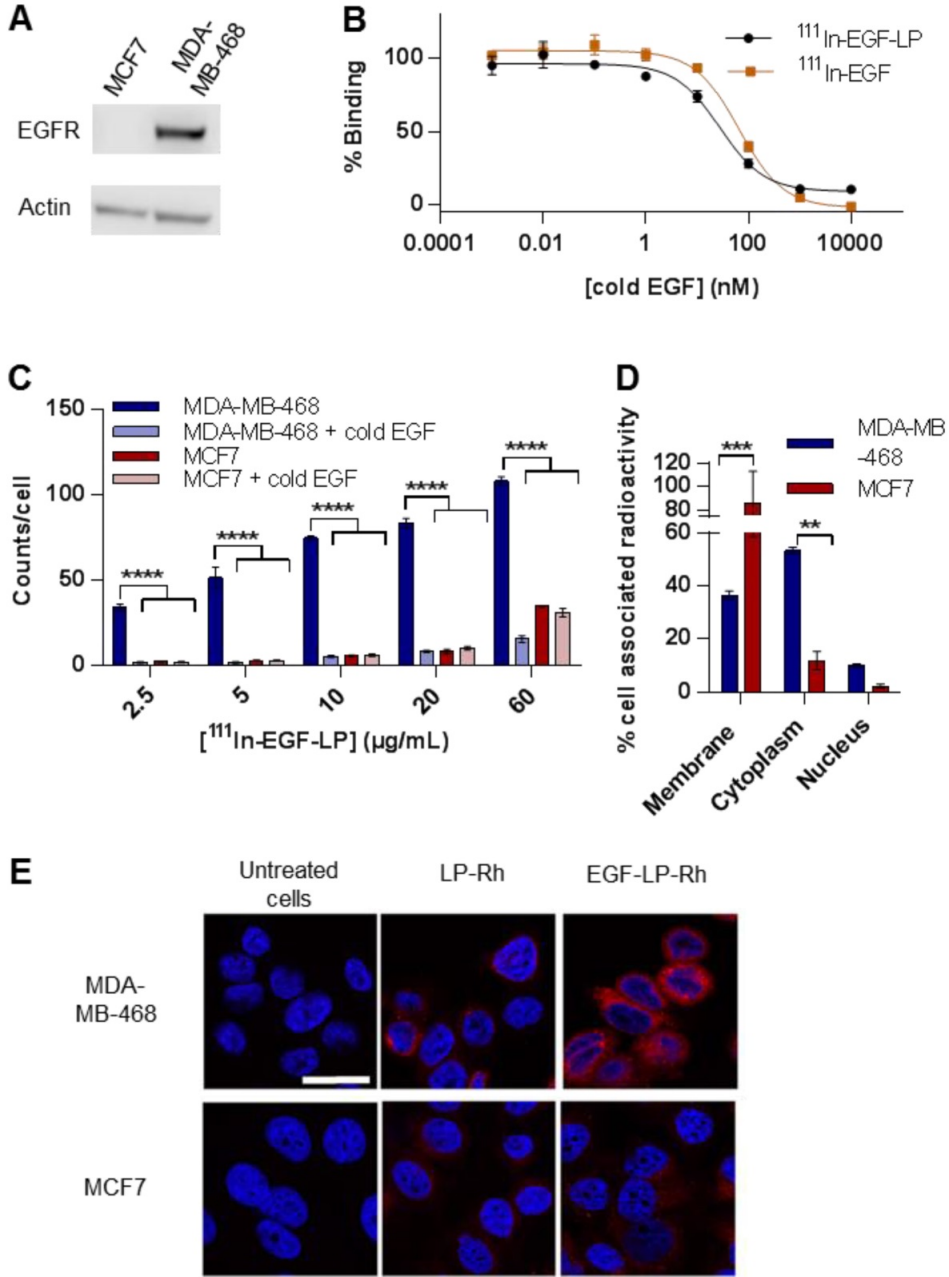
***In vitro* selectivity for EGFR overexpressing cells and subcellular distribution of ^111^In and Dox. (A)** Western Blot characterising the expression level of EGFR in MDA-MB-468 and MCF7 cells. **(B)** Competition binding experiments of both ^111^In-EGF-LP and ^111^In-EGF to MDA-MB-468 cells when treated with increasing concentrations of non-labelled EGF (cold EGF). Incubation 2 h at 4 °C in PBS (n = 3, standard deviation shown, curve fit by nonlinear regression using Graphpad Prism). **(C)** Uptake of ^111^In-EGF-LP by EGFR-positive MDA-MB-468 or EGFR-negative MCF7 breast cancer cells with or without the co-incubation of cold EGF. Incubation 2 h at 37 °C (n = 3, standard deviation shown), **** = p<0.00005 using ANOVA with Bonferroni analysis). **(D)** Intracellular distribution of ^111^In within MDA-MB-468 and MCF7 cells following exposure to ^111^In-EGF-LP, incubation for 2 h at 37 °C (n = 3, standard deviation shown, *** = p<0.0005 and ** = p<0.005 using ANOVA with Bonferroni analysis). **(E)** Visualisation of cellular uptake of rhodamine-containing liposomes by MDA-MB-468 and MCF7 cells using confocal microscopy. Blue = DAPI, red = rhodamine. Images were processed using ImageJ software. Original magnification: 60x (scale = 25 µm).

The binding ability and selectivity of ^111^In-EGF for EGFR was investigated using MDA-MB-468 overexpressing cells (1 x 10^6^ EGFR/cell) and MCF7 (1 x 10^4^ EGFR/cell) as a control (see Figure 2A) 13. To enable these studies, the toxicity of the liposomes had to be minimised, and so empty liposomes (no Dox) were used.

To confirm the binding affinity of ^111^In-EGF-LP to MDA-MB-468, a competition binding study was performed (Figure 2B). Decreased liposome binding to MDA-MB-468 cells was observed with increasing concentrations of competing unlabelled EGF. Notably, similar IC50 values were obtained for ^111^In-EGF and ^111^In-EGF-LP (log(IC50) = 1.82 ± 0.04 and 1.42 ± 0.07 respectively), indicating that the affinity for EGFR is not reduced by grafting EGF on to liposomes.

Uptake studies (Figure 2C) demonstrated statistically significantly higher uptake of radiolabelled EGF-tagged liposomes into MDA-MB-468 compared to MCF7 cells (p<0.00005). The amount of internalised radioactivity was 15.1 ± 1.8% for MDA-MB-468 compared to 1.8 ± 0.3% for MCF7. The greater uptake of EGF-tagged particles is consistent with previous work showing greater internalisation of EGF-tagged gold nanoparticles into MDA-MB-468 cells compared to MCF7 cells 33. Furthermore, the co-incubation of non-radiolabelled EGF with ^111^In-EGF-LP decreased the uptake of liposomes in MDA-MB-468 to the level obtained for MCF7 cells. These results suggest that the liposomes are mainly internalised through EGFR targeting with a small amount of non-specific uptake when added at the high concentrations.

Selectivity of ^111^In-EGF-LP uptake is an important attribute but it is necessary for the radioactivity to be translocated to the nucleus to induce the desired cytotoxic effect. To probe this, the *in vitro* subcellular distribution of ^111^In following incubation of ^111^In-EGF-LP with MDA-MB-468 and MCF7 cells was investigated (see Figure 2D). A statistically (p<0.005) significantly greater (~5-fold) amount of radioactivity was associated with the cytoplasm of MDA-MB-468 cells compared to the cytoplasm of MCF7 cells (53.3 ± 1.2 % versus 11.8 ± 3.4 % of the internalised radioactivity, respectively). More than 85% of the cell-associated radioactivity was found in the membrane fraction of MCF7 cells, indicating non-specific interaction and absence of a specific cell entry mechanism. Greater amounts of radioactivity were also recovered from the nuclei of MDA-MB-468 cells compared to MCF7 cells (10.1 ± 0.6 % vs 2.2 ± 0.7 % of the internalised radioactivity corresponding to 1.5 ± 0.1 % and 0.04 ± 0.01 % of the total radioactivity added to the cells). These findings support the strategy of using ^111^In, since efficient nuclear accumulation and the short-range of the emitted Auger electrons are expected to result in little toxicity in non-targeted cells compared to radionuclides that emit longer range β-particles. Results are also in line with previous work on EGF-tagged PLGA nanoparticles where 5.1 ± 0.1% of the total cell-internalised radioactivity was found in the nuclei of EGFR-overexpressing oesophageal cancer cells 32.

In this study, US-induced cavitation is used to increase the extravasation of the liposomes from the bloodstream to the tumour by increasing blood vessel permeability and establishing convective flow. The liposomes were designed to be stable to US-induced cavitation allowing co-delivery of ^111^In and Dox. Therefore, the direct application of US to cells in the presence of SV is not intended to and does not alter cellular internalisation or subcellular localisation of ^111^In-EGF-LP. This is shown in Supplementary Material and Methods and Figure S2, whereby cell uptake and nuclear localisation of ^111^In-EGF-LP was solely determined by the EGFR status of the cells and the application of US had little effect. This indicates that cavitation-mediated sonoporation does not contribute to cell uptake or cell death in these studies.

Finally, the subcellular localisation of the liposomal contents was visualised by confocal microscopy by replacing Dox with rhodamine (Figure 2E and Figure S3). Greater intracellular fluorescence was observed for EGF-tagged liposomes than for non-functionalised liposomes highlighting again the utility of surface functionalisation for drug delivery. Furthermore, concentration of the dye was observed in the perinuclear space of MDA-MB-468 cells, whereas fluorescence was very faint and distributed throughout the MCF7 cells indicating that these cells may have shown only modest and non-specific uptake of the liposomes. The efficient localisation of EGF-tagged particles within the perinuclear space of EGFR-overexpressing cells is in agreement with results obtained for EGF-tagged gold nanoparticles 33. Flow cytometry was also performed after incubation of MDA-MB-468 and MCF7 cells with rhodamine-containing liposomes (Figure S4 and Supplementary Materials and Method). This confirmed higher uptake of the EGF-LP-Rh in MDA-MB-468 compared to MCF7 cells and that, in MDA-MB-468 cells, EGF-LP-Rh was more efficiently internalised than LP-Rh. These data indicate that the strategy of using US-stable LPs enhances the chance of exploiting the additive effects of ^111^In and Dox since it results in co-localisation of the radionuclide and the agent encapsulated by the liposome.

### ^111^In-EGF-LP-Dox is cytotoxic to and causes DNA damage in EGFR overexpressing cells

Having demonstrated the ability of EGF-mediated targeting to enhance cell uptake and provide favourable intracellular distribution of liposomes, experiments were performed to investigate cancer cell-kill capacity. First, MDA-MB-468 and MCF7 cell lines were treated with EGF-tagged liposomes lacking both drug and radioisotope (EGF-LP) up to a concentration of 0.5 mg/mL. The MTT assay showed that there was no significant increase in cell death compared to controls (SI, Figure S5). This indicates that the liposomes themselves are not cytotoxic.

**Figure 3:**
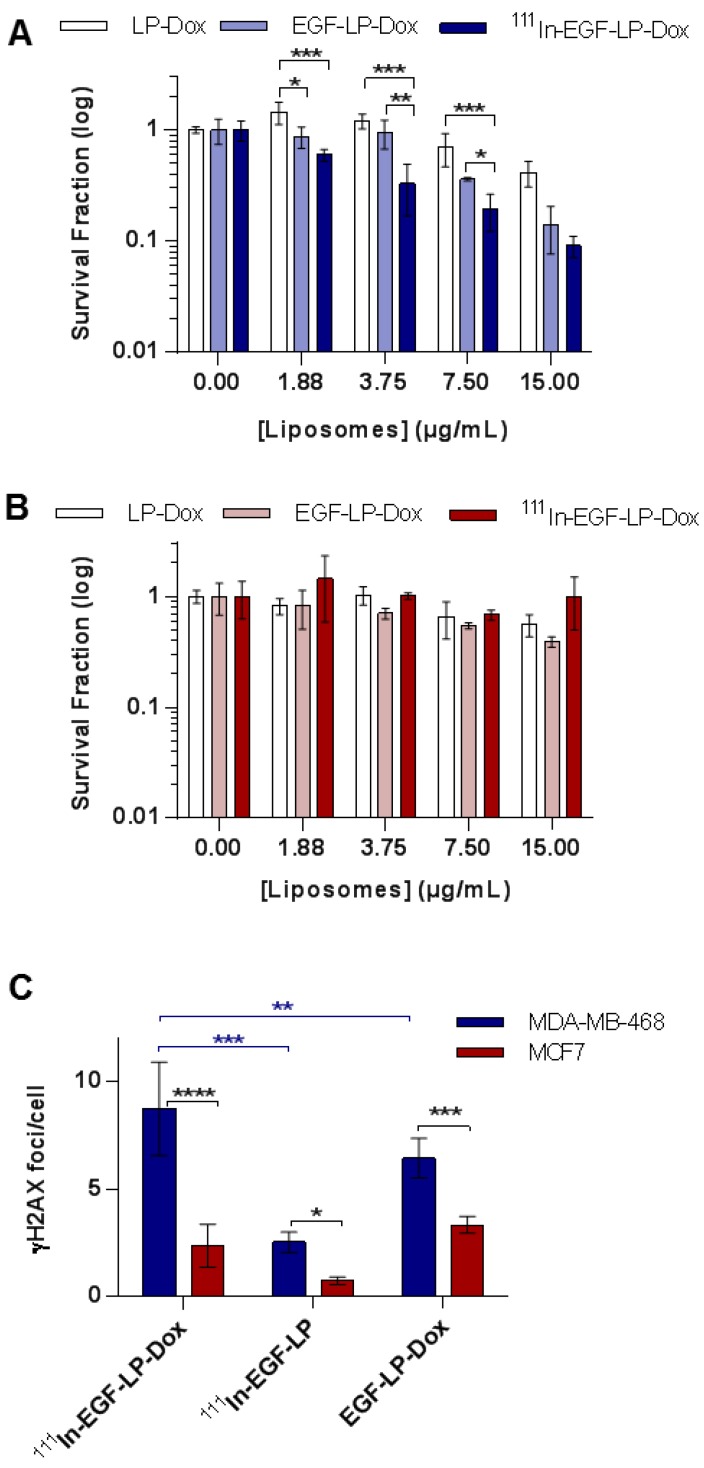
***In vitro* toxicity and DNA damage achieved by ^111^In-EGF-LP-Dox**. Clonogenic studies of **(A)** MDA-MB-468 and **(B)** MCF7 cells following a 24 h treatment and 14 days incubation. (n = 3, standard deviation shown). Statistical analysis was performed using ANOVA with Bonferroni analysis. For each concentration, significant differences between each group are represented, the other comparison were non-significant (* = p < 0.05; ** = p < 0.005 and *** = p < 0.0005). **(C)** DNA damage studies for MDA-MB-468 and MCF7 after treatment with liposomal formulations. Number of γH2AX foci/cell counted using ImageJ software. Incubation for 24 h at 37 °C (n = 3, standard deviation shown **** = p<0.00005, *** = p<0.0005 and ** = p<0.005 using ANOVA with Fisher's LSD analysis), supporting images can be found in SI on Figure S7.

The cytotoxicity of ^111^In-EGF-LP-Dox was then assessed in clonogenic assays in MDA-MB-468 and MCF7 cells (Figure 3A-B and Figure S6). For MDA-MB-468, a substantial decrease in colony formation was observed with increasing concentration of ^111^In-EGF-LP-Dox. At a liposome concentration of 15 µg/mL MDA-MB-468 colony formation was decreased by 3-fold or 5-fold following exposure to EGF-LP-Dox or ^111^In-EGF-LP-Dox respectively, compared to LP-Dox (Figure 3A). At a concentration of 3.75 and 7.50 µg/mL, a statistically significant difference was observed between EGF-LP-Dox and ^111^In-EGF-LP-Dox. These results highlight the value of combining Dox and ^111^In-EGF in one construct to give combined chemo-radionuclide therapy and the additive effect of the two entities on MDA-MB-468 cells. There was a decrease in MCF7 colony formation upon treatment with increasing concentrations of Dox-containing liposomes: LP-Dox, EGF-LP-Dox and ^111^In-EGF-LP-Dox. However, the effect was less pronounced in MCF7 than in MDA-MB-468. For example, more than 40% of MCF7 cells survived following treatment with ^111^In-EGF-LP-Dox containing 15 µg/mL Dox while only 8% of MDA-MB-468 colonies survived these conditions.

Having established the benefit of the targeted combined delivery of radioisotope and chemotherapeutic agents, the mechanism of action was then addressed by measuring DNA damage. As presented in Figure 3C and Figure S7, the formation of γH2AX foci was quantified as it is a well-established marker of the DNA damage response 47. All ^111^In or Dox containing treatments caused induction of γH2AX in both cell lines although H2AX foci were formed to a lesser extent in MCF7 cells compared to MDA-MB-468 cells. In MDA-MB-468, the number of γH2AX foci was substantially higher after treatment with ^111^In-EGF-LP-Dox (8.7 ± 2.2 foci/cell) than with ^111^In-EGF-LP (2.5 ± 0.5 foci/cell) or EGF-LP-Dox (6.4 ± 0.9 foci/cell).

Therefore, the data presented in Figures 2 and 3 confirm the feasibility of using radiolabelled Dox-containing liposomal drug carriers surface-modified with EGF for selective chemo-radiotherapy in an *in vitro* setting.

### US-mediated extravasation was shown using a gel vessel phantom

**Figure 4:**
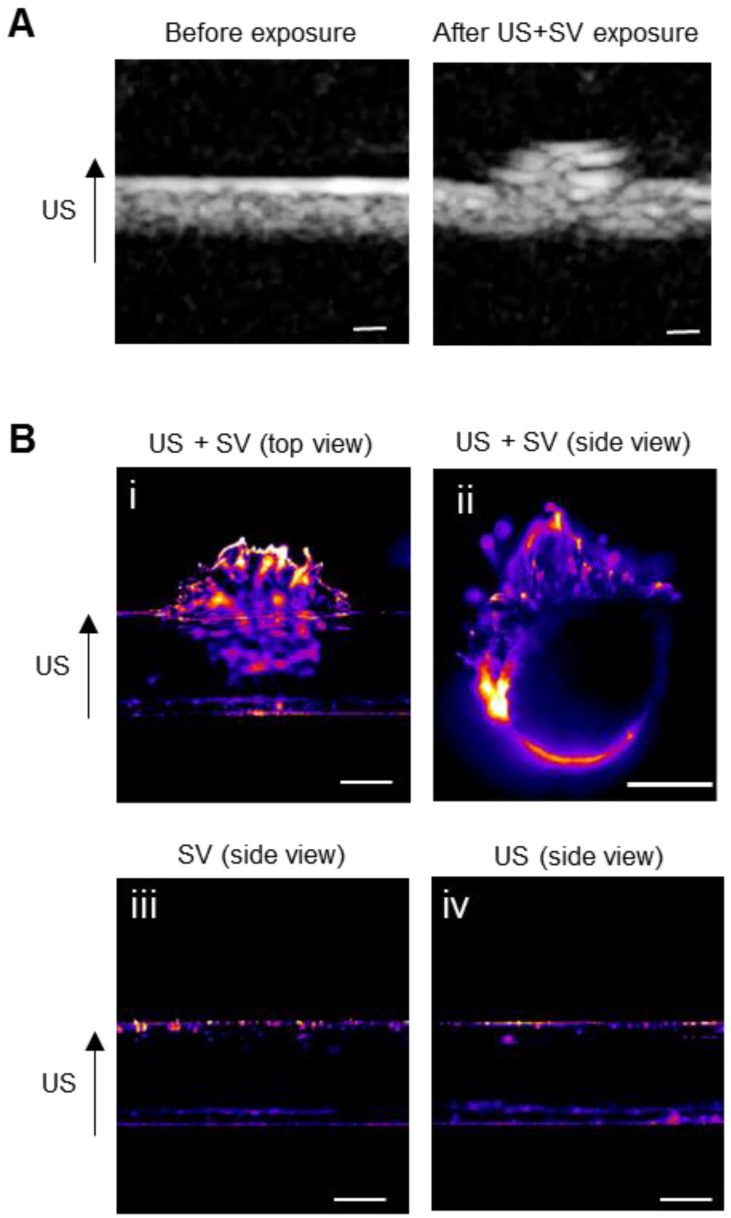
**Ultrasound mediated cavitation for improved extravasation of the liposomal formulation in a gel phantom**. **(A)** B‐mode images of the flow channel before and after exposure to EGF-Rh-LP, US and SV. Scale: 500 µm. **(B)** Fluorescence in the agarose channel following administration of EGF-Rh-LP, SV and US (top and side views, panels (i and ii), or EGF-Rh-LP and SV only (side view, panel iii) or EGF-Rh-LP and US only (side view, panel iv). Scale: 500 µm.

To evaluate the potential of SV for US-mediated enhanced delivery of ^111^In-EGF-LP-Dox, a blood vessel phantom was used (see Figure S8). It is composed of channels created in an agar gel with a pore diameter of 500 nm 48, similar to the size of gaps between endothelial cells in tumour tissues. EGF- and rhodamine-tagged liposomes (EGF-Rh-LP) were introduced into and flowed through the channels with or without SV and US application. Conventional B-mode images were captured (at low Mechanical Index to avoid destruction of bubbles) during the excitation of SV to show extravasation in real time (Figure 4A). After excitation, the channels were excised and imaged by fluorescence microscopy (excitation at 545 nm and emission at 620 nm) to detect rhodamine (Figure 4B). Extravasation of the EGF-Rh-LP was clearly seen when US was applied in the presence of SV (Figure 4B, panels i and ii). On the contrary when the EGF-Rh-LP was introduced into the channel with SV but without US (Figure 4B, panel iii) or with US but without SV (Figure 4B, panel iv), no extravasation was detected which is in agreement with published reports 40, 49.

### Stability, biodistribution and pharmacokinetic profile of ^111^In-EGF-LP were evaluated *in vivo* using MDA-MB-468 tumour bearing mice

**Figure 5:**
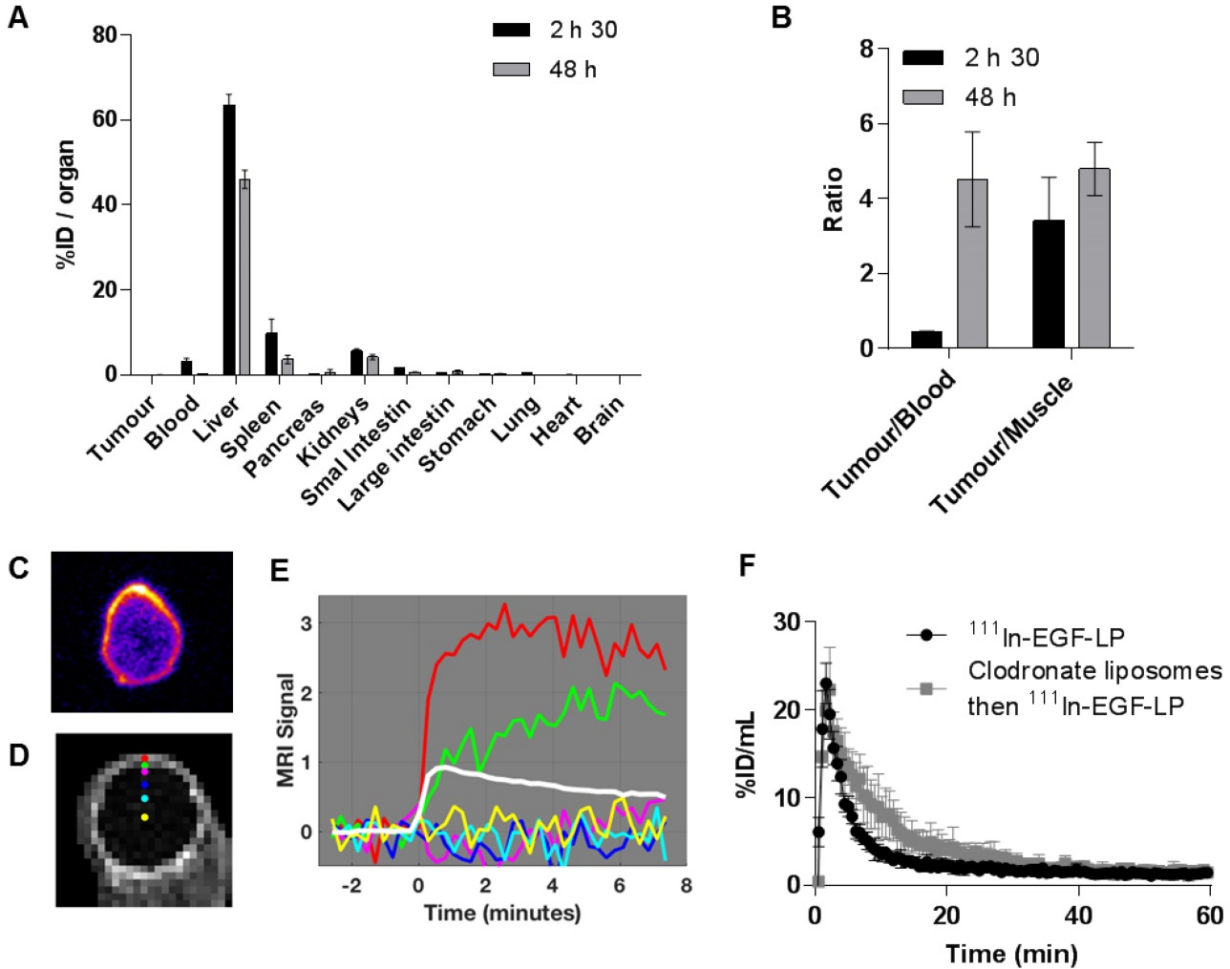
** Biodistribution and pharmacokinetic profile of the ^111^In-EGF-LP liposomal formulation *in vivo* in mice bearing subcutaneous MDA-MB-468 tumours. (A)** Biodistribution of ^111^In-EGF-LP in mice bearing MDA-MB-468 xenografts expressed as percentage of injected dose per organ (%ID/organ) (n = 4, standard deviation shown). **(B)** Ratios of tumour to blood and muscle. **(C)** Autoradiography showing the distribution of ^111^In in one slice of the tumour (representative image). **(D)** MRI section of an MDA-MB-468 tumour and (E) MRI signal enhancement obtained after injection of a MRI contrast agent to mice bearing MDA-MB-468 tumour. The curve for muscle in white is shown for comparison. **(F)** Blood lifetime of ^111^In-EGF-LP obtained by SPECT with or without pre-treatment of mice with clodronate liposomes (n = 3, standard deviation shown).

The biodistribution of ^111^In-EGF tagged liposomes was studied in athymic nude mice bearing MDA-MB-468 xenografts (Figure 5A-B). Values are given as a percentage of the injected dose per organ or per gram of organ (%ID/organ and %ID/g) in SI (Table S1 and S2). As is typical for a liposome formulation, radioactivity was mainly detected in the liver followed by the spleen. The Tumour/Muscle ratio (3.4 ± 1.2 at 2.5 h and 4.8 ± 0.7 at 48 h) indicates differential uptake in tumour versus healthy tissue likely due to the EPR effect and binding to EGFR in the tumour. However, the levels recovered from the blood and tumour are much lower than expected. Indeed, other EGF-tagged nanoparticles have shown higher tumour uptake (%ID/g ~ 1 at 24 h) in different tumour models 32, 50. Crucially, in this case, the radioactivity was also mainly distributed in the periphery of the tumour as shown by autoradiography (Figure 5C).

To investigate whether this intratumoural distribution was related to poor vascularisation of the tumour, the perfusion of MDA-MB-468 xenografts was studied using Dynamic Contrast-Enhanced Magnetic Resonance Imaging (DCE-MRI) after injection of a MRI contrast agent as shown on Figure 5D-E. These data suggest that perfusion is restricted to the rim of the tumour (red line in Figure 5E). The contrast enhancement shown as a green line (Figure 5E) is consistent with the contrast agent leaking out of the vessels in the rim and diffusing into the surrounding tissue. Beyond that (magenta, blue, cyan and yellow), no enhancement was detected during the experiment suggesting that the range of the diffusion is relatively short. The average signal enhancement curve for muscle is shown in white for comparison. The signal in the rim of the tumour is much higher than that of the muscle and continues to increase after the muscle signal has reached its maximum. This suggests a dense leaky vasculature in the xenograft, very different to normal tissue 51. The poor vascularisation of the MDA-MB-468 tumour model would be expected to prevent accumulation of ^111^In-EGF-LP in the tumour and explains why the radioactivity is mainly located in the periphery of the tumour.

Degradation of the liposomes and consequent release of free ^111^In-EGF, which has been shown to be cleared quickly (τ_1/2_ < 30 min 16), is not the cause of the rapid clearance from the bloodstream seen in this study. Indeed, an *in vivo* stability study was performed and showed no evidence of degradation of ^111^In-EGF-LP 2.5 h after i.v. injection (see Figure S9). The fast clearance of the liposomes is more likely to be due to their rapid sequestration in the liver possibly due to EGF-mediated binding to hepatocytes. Expression of EGFR together with the extensive perfusion of this organ, would lead to a more marked uptake than in the tumour 50, 52. To investigate this further, the pharmacokinetic profile of ^111^In-EGF-LP was determined *via* SPECT imaging for 2 h with or without pre-treatment with clodronate liposomes (Figure 5F). In liposomal form, clodronate is taken up by macrophages in the liver (Kupffer cells), spleen and bone marrow and causes apoptosis. It has been shown that macrophage depletion is complete 24 h after intravenous injection of clodronate liposomes, which are therefore expected to decrease macrophage-mediated blood clearance of ^111^In-EGF-LP 41, 42. However, for both clodronate treated and non-treated groups of mice,^ 111^In-EGF-LP was rapidly cleared from the blood with a half-life of 5-8 min. This differs greatly from the results obtained for Doxil^®^, a long-circulating non EGF-tagged liposome formulation, which has a distribution half-life of approximately 27 h in rat models 53. These findings indicate that the fast clearance of ^111^In-EGF-LP is due to direct EGFR targeting of the liver rather than phagocytosis of liposomes by macrophages. One strategy to prevent hepatic sequestration of EGF-tagged nanoparticles would be to encapsulate both Dox and ^111^In-EGF in US labile liposomes, a strategy which is currently under investigation. Despite the rapid clearance from the circulation of ^111^In-EGF-LP, the application of US-mediated cavitation has the potential to increase uptake into the tumour.

### US-mediated cavitation of SV increases the tumour uptake of ^111^In-EGF-LP

**Figure 6:**
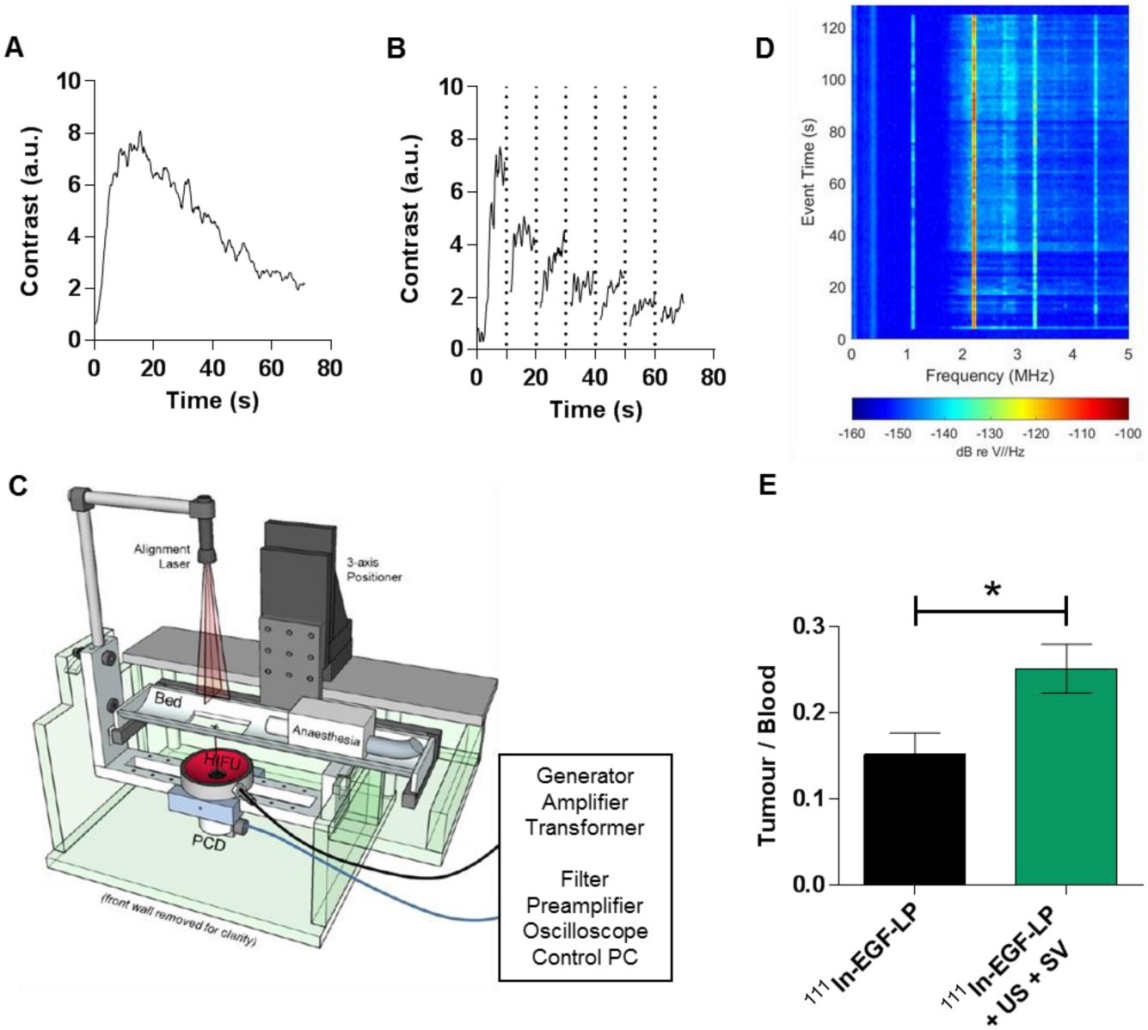
***In vivo* impact of the US induced cavitation on the distribution of the ^111^In-EGF-LP liposomal formulation. (A)** US signal in MDA-MB-468 tumour after injection of SV. **(B)** US signal in the tumour after injection of SV and high amplitude ('flash') US exposures every 10 s (vertical dots line).** (C)** Schematic of the US setup used for delivering US *in vivo*. **(D)** Acoustic data collected during US/SV treatment (representative results for one mice), showing harmonic and ultraharmonic bubble scattering, as well as elevated broad spectrum noise.** (E)** Ratio Tumour/Blood obtained 10 min post injection of ^111^In-EGF-LP (n = 3, standard deviation shown, p = 0.0167 using unpaired two-tails test, non-parametric).

US mediated delivery was studied *in vivo* in mice bearing MDA-MB-468 tumours. Values of pulse repetition period and total exposure time were determined from perfusion experiments where tumours were imaged as a function of time after an injection of SV (Figure 6A). A time period of 45 s was identified as the point at which the mean tumour contrast enhancement dropped to half maximum level. A destruction-replenishment experiment was conducted separately with high amplitude ('flash') exposures every 10 s to observe how quickly the tumour contrast reached a local plateau. Review of all replenishment data sets (an example is shown in Figure 6B) suggested a minimum pulse repetition period of 1.2 seconds. Pulse length was guided by radiation force/extravasation considerations as reported in Mannaris *et al*. 49, where the benefit of extending the length beyond a few thousand cycles was minimal, likely because flow rate was insufficient to replenish the supply of microbubbles in the US focal region. Given the above constraints, pressure amplitude was chosen to maximize radiation pressure (quadratic in pressure amplitude) for extravasation and transport while avoiding tissue damage both in the focus and off target. A range of peak negative pressures up to 2.2 MPa was tested in an *in vivo* pilot study of tumour-bearing mice both with and without SV. While no evidence of adverse effects was found at any pressure, a final value of 2.0 MPa was chosen to provide an extra margin of safety. The device design for US exposure *in vivo* is presented in Figure 6C.

To study the effect of US-mediated cavitation on ^111^In-EGF-LP delivery to tumour, mice received either ^111^In-EGF-LP only or ^111^In-EGF-LP, US and SV. The success of SV delivery and cavitation instigation in the tumour are presented in Figure 6D. It shows harmonic and ultraharmonic (integer and half-integer multiples of the 1.1 MHz FUS drive frequency) bubble scattering, as well as elevated broad spectrum noise (light blue fill between 2-5 MHz) suggesting inertial cavitation 29. These cavitation events provide useful feedback on the course of the procedure and are obtained non-invasively which is of interest for US as a delivery tool.

The combination US plus SV was sufficient to significantly increase the uptake of ^111^In in tumour in comparison to the control (Figure 6E). Indeed, the Tumour/Blood ratio 10 min p.i. was 0.15 ± 0.03 for mice receiving ^111^In-EGF-LP only while it was 0.25 ± 0.03 for mice receiving ^111^In-EGF-LP + SV + US (p = 0.0167). These results show promise for the use of physical stimuli for enhanced delivery of radiopharmaceuticals, a field that had been under-investigated until now. However, the radioactivity has mainly distributed in the periphery of the tumour as shown by autoradioagraphy (see SI, Figure S10) and the level of tumour uptake with US was lower than in some previous reports. For example, Wang *et al.* found that the delivery of polymeric nanoparticles was 4 to 14-fold higher in colon cancer xenografts when US was applied 26. Bazan-Peregrino *et al* 54 and R. Carlisle *et al*. 55 worked on oncolytic adenovirus and found up to 50 or 30-fold increases respectively in tumour infection in mice bearing human xenograft tumours. It has to be noted that in this case the initial infection with the oncolytic virus was probably lower making it difficult to draw a direct comparison with our study. There are two possible explanations for the modest increase in drug delivery achieved through the application of US in this study. It is possible that cavitation occurred at the periphery of the tumour, but that the strength of individual collapse events and/or the number of bubbles present was insufficient to propel the liposomes very far. US safety concerns limited what could be achieved as we could not arbitrarily continue to increase the incident pressure without risking tissue damage. The poor vascularity of the tumour also further restricted the extent to which liposomes could be distributed by any means, including cavitation. We hypothesize that poor vascularity severely limited the proportion of the tumour volume that contained microbubbles and was therefore likely the major determinant of poor uptake and the explanation for why radioactivity was confined to the periphery of the tumour. Nevertheless, all these reports emphasize the interest of cavitation-enhanced delivery for a wide range of therapeutic agents. Such a strategy has the potential to solve the common problem of poor penetration of drug into tumour and could easily be implemented for a range of solid tumours.

One limitation of these studies is related to the tumour model used (murine xenografts). While these tumours can be easily targeted with US, it may not always be the case for patients in clinic. Better *in vivo* or *ex vivo* models, more representative of the morphology and US access, need to be developed to gain better knowledge and to optimise the use of cavitation to enhance drug delivery. The development of strategies to increase the duration of US-induced cavitation should be performed in parallel in order to sustain and improve delivery over longer periods and to maximise the effect of cavitation for drug delivery. The injection of multiple boluses or a continuous infusion of cavitation agent over a longer period of time, in addition to the development of new cavitation agents that could match the pharmacokinetic profile of the therapeutic agents studied will be investigated in future work.

## Conclusion

In the current study, a new liposome, ^111^In-EGF-LP-Dox, was designed for chemo-radionuclide therapy of EGFR-positive cancer and the use of US-induced cavitation to enhance its delivery to breast cancer tumour models was investigated. The liposomal formulation has shown promise *in vitro* in terms of EGFR-targeted cellular uptake for drug delivery as well as for therapeutic effect. This report also provides evidence that US-mediated cavitation can enhance delivery in a tissue-mimicking phantom and more importantly *in vivo.* This is promising for the delivery of radiopharmaceuticals and of great interest as this strategy could easily be implemented for the co-delivery of additional drugs in the treatment of a range of solid tumours. Future work will focus on the optimisation of the liposome design and study of the therapeutic effect induced by the use of cavitation-enhanced delivery. In particular, the system will be tested with cavitation agents that provide more sustained cavitation duration than SV. The extent of tumour vascularisation is also believed to be a determinant of success of cavitation-enhanced delivery and will be studied by testing in well perfused tumour models.

## Supplementary Material

Supplementary figures and tables.

## Abbreviations

DCE-MRI: dynamic contrast enhanced magnetic resonance imaging
DMEM: Dulbecco's modified eagle medium
Dox: doxorubicin
DTPA: diethylenetriaminepentaacetic acid
EGF: epidermal growth factor
EGFR: epidermal growth factor receptor
FBS: fetal bovine serum
FUS: focused ultrasound
ID: injected dose
^111^In: indium-111
i.v.: intravenous
LP: liposome
MUTA: murine ultrasonic therapy apparatus
PCD: passive cavitation detector
p.i.: post injection
Rh: rhodamine
SAT: system for acoustic transfection
s.c.: subcutaneous
SF: surviving fraction
SI: supplementary information
SPECT: single photon emission computed tomography
SV: Sonovue®
US: ultrasound
