# Supplementary material for: Ultrasound-mediated cavitation enhances the delivery of an EGFR-targeting liposomal formulation designed for chemo-radionuclide therapy
Source: Theranostics. 2019 Jul 28;9(19):5595–609. doi: 10.7150/thno.34669 (PMC6735398; doi:10.7150/thno.34669)
Supplement: Supplementary file 1 — Supplementary figures and tables. [file thnov09p5595s1.pdf]

Supplementary Information

Ultrasound-mediated cavitation enhances the delivery of an EGF-targeted liposomal formulation designed for chemo-radionuclide therapy

Eloise Thomas[1], Jyothi U. Menon[1], Joshua Owen[2], Irini Skaripa-Koukelli[1], Sheena Wallington[1], Michael Gray[2], Christophoros Mannaris[2], Veerle Kersemans[1], Danny Allen[1], Paul Kinchesh[1], Sean Smart[1], Robert Carlisle[2*], Katherine A. Vallis[1*].

[1] CRUK/MRC Oxford Institute for Radiation Oncology, Department of Oncology, University of Oxford, Oxford, OX3 7DQ, UK.

[2] Institute of Biomedical Engineering, Department of Engineering Science, University of Oxford, Oxford, OX3 7DQ, UK.

*These authors contributed equally to the work.

Supplementary Material and Method

^{111}In-EGF-LP-Dox characterisation

The size and zeta potential of the liposomes were determined by Dynamic Light Scattering (DLS) (ZetaSizer Nano, Malvern Instruments Ltd, Malvern, Worcestershire, UK) in water containing 10 mM NaCl at pH = 7. In the case of ^{111}In-EGF-LP-Dox, the sample was stored until the radioactivity had decayed for measurement at a later time. As described in literature, Dox content was determined by absorbance measurements at 480 nm [1] and its release was studied by dialysis and fluorescent measurements [2] for an excitation at 480 nm and emission at 560 nm (TECAN Infinite 200 PRO plate reader, Tecan Group Ltd., Männedorf, Switzerland). The amount of EGF present on the liposomes was determined using Human EGF Quantikine ELISA kit (R&D Systems, Inc., Minneapolis, MN, USA). Radiolabelling yield was determined by silica thin-layer chromatography (TLC) in 0.1 M sodium citrate buffer (pH 5.0) and radioactivity measurements of the silica strips using a AR-2000 radio-TLC scanner (Eckert & Ziegler Radiopharma, Inc., Hopkinton, MA, USA). Purity of the liposomes batches (> 90 %) was determined by eluting ^{111}In-EGF-LP-dox on a G75 column with a 0.1 M sodium citrate buffer (pH 5.0) and measurement of the aliquots radioactivity using an automated Wizard gamma counter (Perkin Elmer, Waltham, MA, USA).

Formulation stability at 4 °C (storage conditions) and 37 °C (diluted in PBS) was evaluated over one month. No significant change was detected in size, polydispersity index, zeta-potential or ^{111}In complexation by DTPA-EGF but 15% and 45% release of ^{111}In-EGF from liposomes was detected 4 days and 1 month after synthesis, respectively. Therefore, only freshly prepared liposomes where used for *in vitro* and *in vivo* experiments.

Stability under ultrasound (US)

EGF-LP-Dox (350 or 60 µg/mL) and SonoVue® microbubbles (SV, 0.25 mg/mL, Bracco Diagnostics Inc) were added to 35 mm, high µ-Dishes (ibidi GmbH, Am Klopferspitz, Planegg/Martinsried). Dishes were exposed to US (1.1 MHz, 2.0 MPa, 3 kcycles, 1.2 pulse per repetition) for 45 s using the System for Acoustic Transfection (SAT) chamber already described [3]. SAT was equipped with a focused US (FUS) transducer (H102, Sonic Concepts, Bothell, WA, USA) of fundamental frequency 1.1 MHz. Experiments were performed in triplicate and solutions of liposomes analysed as detailed in the previous paragraph.

Effect of US-induced cavitation on cellular uptake and subcellular localisation of ^{111}In-EGF-LP

MDA-MB-468 and MCF7 cells were seeded in 35 mm, high µ-Dishes with a glass base (ibidi GmbH, Am Klopferspitz, Planegg/Martinsried) and left to attached for 36 h (3.10^5 cells/dish). Dishes were exposed to ^{111}In-EGF-LP only (5 µg/mL, 0.15 MBq/mL), or ^{111}In-EGF-LP, SV (25 µg/mL) and US using the SAT device as presented above. Cells were incubated for 2 h at 37°C. The radioactivity associated with different cellular compartments was determined using Nuclei EZ Prep Nuclei Isolation Kit (Sigma-Aldrich, Dorset, UK) as described in the manuscript. Protein content was then determined using a BCA protein assay (ThermoScientific, Massachusetts, USA).

Flow cytometry

MDA-MB-468 and MCF7 cells were seeded in six-well plates with a density of 0.25×10^6 cells/well and incubated overnight to allow adhesion of cells. The original culture medium was then replaced by fresh supplemented medium, and cells were incubated with rhodamine-containing liposomes (LP-Rh or EGF-LP-Rh). After 24 hours of incubation, cells were washed twice with PBS, collected by trypsinisation, pelleted, washed in 0.5 ml of PBS and resuspended in 0.2 ml of PBS. The cell suspension obtained was analyzed by Flow Cytometric Analysis (FACSort BD Biosciences, California, USA). A total of 20,000 events were acquired for each sample. FlowJo software (Tree Star Inc., Oregon, USA) was used for the analyses.

MTT assay

In vitro studies were conducted to study cell viability following uptake of the EGF-LP. MDA-MB-468 and MCF7 cells were seeded in a 48-well plate at a density of 8.5 x 10^3 cells.cm^{-2}. Following incubation for 24 hours, the cells were treated with EGF-LP for 2 hours. After incubation, cells were washed twice with PBS and fresh medium was added. The cells were incubated for a further 4 days and then used in MTT assays.

In vivo studies: ethical statement

All procedures were conducted in accordance with the Animals Scientific Procedures Act of 1986 (UK) (Project License Number 30/3115 and P13B66CD9 issued by the Home Office) and protocols approved by the Committee on the Ethics of Animal Experiments of the University of Oxford. Mice were housed (n = 4-6 per cage) in individual ventilated polycarbonate solid-bottomed cages. Airflow within the cages was on a positive pressure and was controlled electronically by an IVC air handling system (Techniplast UK). A 12 h dark and light cycle was implemented with the ambient air temperature set at 21 ± 2 °C with 55 ± 10% humidity. All animals were provided with certified rodent diet, filtered water ad libitum, autoclaved bedding and nesting material and cage enrichment. All anaesthesia sessions were performed under 4% (induction) or 2% (maintenance) isoflurane aesthesia delivered in oxygen enhanced room air and temperature maintain at 35-37°C using heating plate. Throughout imaging experiments, temperature and respiration rate were monitored and all efforts were made to minimize suffering. Where an indwelling cannula was used to administer contrast agents and treatments, a catheter (PE10, 0.28/0.64 mm internal/external diameter; Linton Instrumentation) was inserted into the lateral tail vein.

In vivo stability

Animals were anaesthetised, cannulated and received ^{111}In-EGF-LP (5 MBq, 120 µg of lipids) intravenously (i.v.) followed by 50 µL of saline to flush the cannula. Mice were euthanized at 10 min, 2.5 h, 24 h and 48 h post injection (p.i.). Blood was immediately collected, mixed with heparin and centrifuged for 5 min. The supernatant was immediately assayed using TLC and G75 column as described previously (section "^{111}In-EGF-LP-Dox characterisation", SI).

SPECT imaging

SPECT imaging was performed using the single gantry VECTor4CT system (MIlabs, Utrecht, The Netherlands) fitted with the HE-UHS-RM SPECT/PET collimator (1.8 mm pinholes). Animals (n = 3) were anaesthetised (2 - 4% isoflurane in room air/oxgygen 80/20 v/v), tail vein cannulated and placed into a bespoke rat cradle adjusted for mouse imaging. ^{111}In-EGF-LP (8 MBq, 120 µg) was administered i.v. and mice were imaged dynamically for ~100 min, acquiring 200 frames of 30 s focussed on the heart. Data were acquired in list mode (0 – 1200 keV) using MILabs acquisition software v7.39. Triple-energy-window based scatter and cross-talk correction was applied for each photon peak window (photopeaks: 155.7-190.3 and 222.3-271.7 keV with background windows set to 148.8-155.7 keV, 190.3.8-197.2 keV, 212.4-222.3 keV and 271.7-281.6 keV) and the associated calibration factor was determined to allow quantitation of SPECT images. All images were reconstructed with MILabs reconstruction software v3.24 on 0.8 mm isotropic 3D voxel grids using dual matrix similarity regulated ordered-subset expectation maximization (dual matrix SROSEM) [4]. Following reconstruction, the SPECT and their corresponding CT data were co-registered and re-sampled to equivalent 200 µm voxel sizes. CT based attenuation correction was applied. Reconstructed images were viewed and analysed using PMOD v.3.37 (PMOD Technologies, Zurich, Switzerland). A spherical volume of interest (VOI) was drawn on CT data. To reduce interindividual variation, all data are presented as percentage of the injected dose (MBq) corrected per selected tissue volume (%ID/mL). Whole body CT was performed for anatomical referencing and CT based attenuation correction. Images were acquired at 50 kV and 0.24 mA using continuous rotation (40 degrees/s) and were reconstructed using cone-beam filtered back projection (Feldkamp algorithm) on a 0.2 mm voxel grid. Beam hardening and ring artefacts were corrected, and the voxel numbers were converted into Hounsfield units by using a pre-measured calibration factor

MRI

MRI was performed with a 7.0 T 210 mm horizontal bore VNMRS preclinical imaging system equipped with 120 mm bore gradient insert (Varian Inc, CA) and a 32 mm ID quadrature birdcage coil (Rapid Biomedical GmbH, Germany). DCE-MRI was performed using a cardio-respiratory gated 3D spoiled gradient echo scan with TR 1.6 ms, TE 0.64 ms, FOV 60×30×30 mm^3, matrix 128×64×64, gradient spoiling with 159 mT/m for 0.5 ms in all three axes, RF hard pulse duration 16 µs, FA 5°, and RF spoiling. Data were acquired in blocks of 64 k-space lines and the two data blocks acquired prior to detection of each breath were reacquired immediately after the same breath [ref]. 50 repeats of the 3D scan were performed with 30 µl of a Gd-contrast agent (Omniscan, GE Healthcare) infused via a tail vein cannula over 5 s starting at the beginning of frame 11/50 [5]. Analysis of the DCE data was performed using in-house software written in matlab. 3D maps of the initial area under the curve at 90 seconds (iAUC90) were generated to assess the distribution of perfusion within the tumours.

Contrast Enhanced Ultrasound

Contrast enhanced ultrasound was performed using the Vevo3100 (FUJIFILM Visualsonics, Joop Geesinkweg 140, 1114 AB Amsterdam, Netherlands) using a MX250 probe (13-24 MHz, Centre Transmit: 20 MHz Axial Resolution: 75 µm). Animals were anaesthetised, cannulated and their temperature monitor with a rectal probe. Power Doppler mode has first used to ascertain the optimal focal point within the tumour where perfusion was most prevalent. Once this was achieved, the Non-Linear Contrast mode was selected and the image acquisition started while a bolus of SV (50 µL) was

injected through the indwelling cannula (Frequency: 18MHz, Power: 10%, Frame Rate: 15, Contrast Gain: 30.0dB, Sensitivity: 1, Dynamic Range: 40dB). VevoLab software was used to trace regions of interest within the tumour to visualise the rate of perfusion and determine the time corresponding to the maximum signal into the tumour (t_{max}). 10 min later, a second acquisition was performed by injecting a bolus of SV (50 µL) and applying a "burst" (high amplitude exposures) at t_{max} and every 10 s after.

US-mediated delivery: alignment of the set-up

Prior to all testing, the nested source and Passive Cavitation Detector (PCD) transducers were aligned to a reference mark on the mylar bed based on the timing and strength of reflections from a 1mm diameter target temporarily placed on the mylar bed. Drive signals for alignment were provided by a pulser/receiver (Panametrics 5072, Olympus NDT) and observed with the HS3 digitizer. Once alignment had been established, the target was removed, and a crosshair laser was further aligned to the bed reference mark for use as an optical guide during therapy.

Supplementary figures

Figure S1: **The physico-chemical properties of the liposomal formulation are not modified after exposure to US and SV mediated cavitation**. **(A)** Size obtained by DLS. **(B)** Dox release in PBS at 37 °C, 0.35 mg/mL, (n = 3, standard deviation shown). The Dox release is about 12.5% at 10 h and 50% at 140 h. **(C)** Thin Layer Chromatography obtained after elution in sodium citrate (0.1 M, pH = 5) showing only one peak for ^{111}In-EGF-LP-Dox (radiolabelling yield > 95%, 300 MBq/mg of liposomes). **(D)** Elution on a G75 column (elution with a sodium citrate buffer, 0.1 M, pH = 5) of ^{111}In, ^{111}In-EGF and ^{111}In-EGF-LP-Dox before and after exposure to US and SV. Purity is higher than 90% and 85% for ^{111}In-EGF-LP before and after exposure to US and SV respectively.

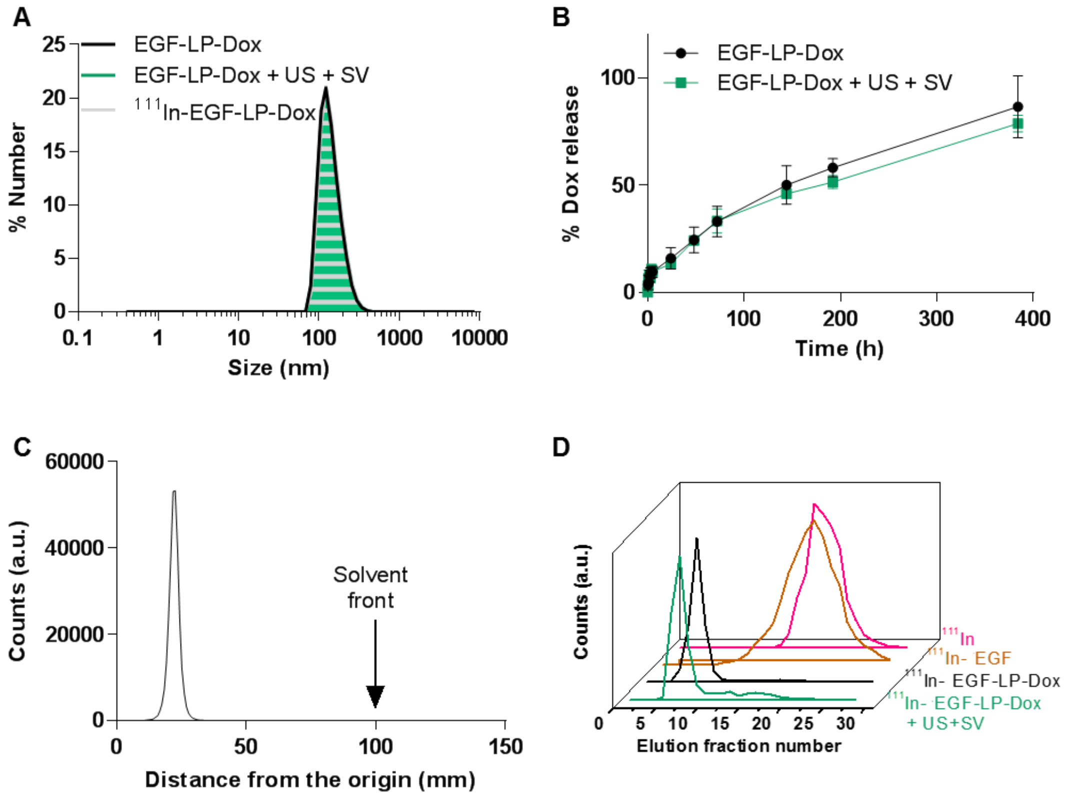

Figure S2: Effect of US-induced cavitation on the uptake and subcellular localisation of ^{111}In-EGF-LP in MDA-MB-468 and MCF7 cells. Results are expressed as counts per minutes (CPM) per µg of protein (n = 4). No significant difference was observed between the groups treated with liposomes, US and SV and the group treated with liposomes only.

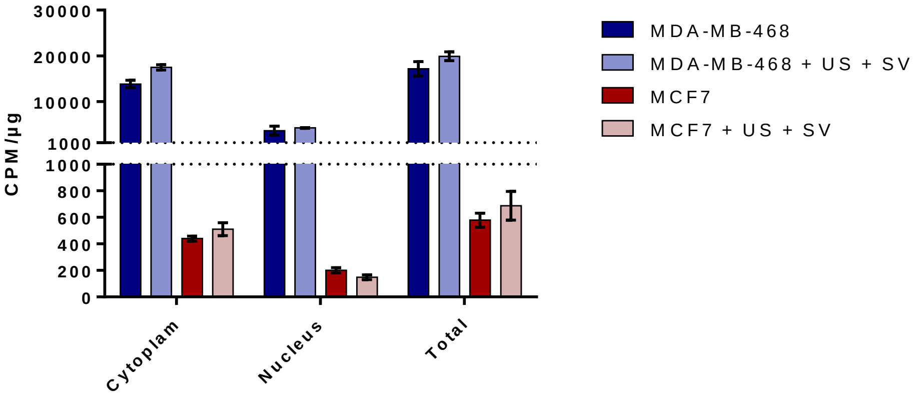

Figure S3: Visualisation of cellular uptake of rhodamine-containing liposomes by MDA-MB-468 and MCF7 cells using confocal microscopy. Blue = DAPI, red = rhodamine. Images were processed using ImageJ software. Original magnification: 60x (scale = 25 μm).

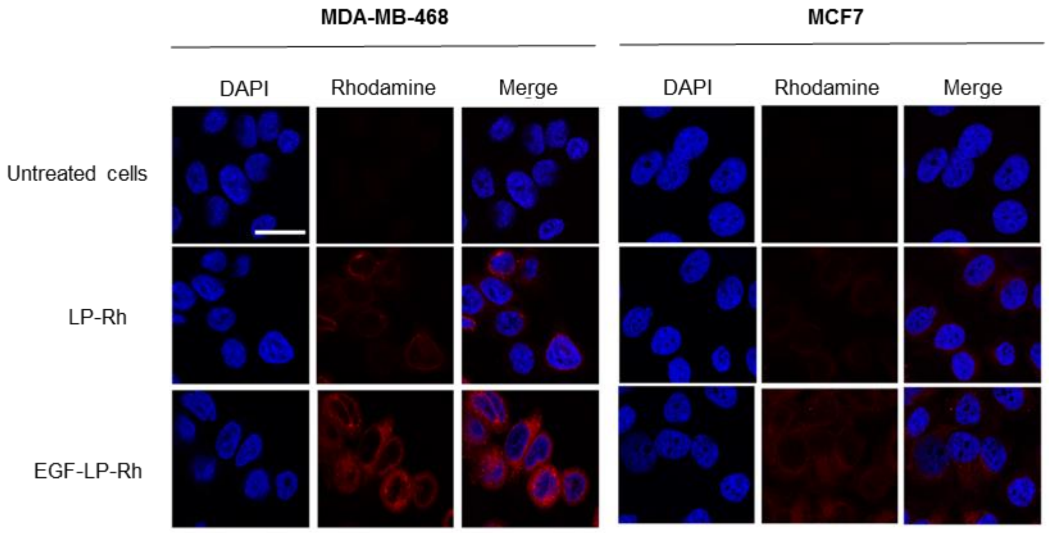

Figure S4: Flow cytometry performed after incubation of MDA-MB-468 and MCF7 rhodamine-containing liposomes. Incubation 24 h at 37 °C. Higher uptake of the EGF-LP-Rh formulation is detected in MDA-MB-468 in comparison to the uptake in MCF7 cells or LP-Rh liposomes

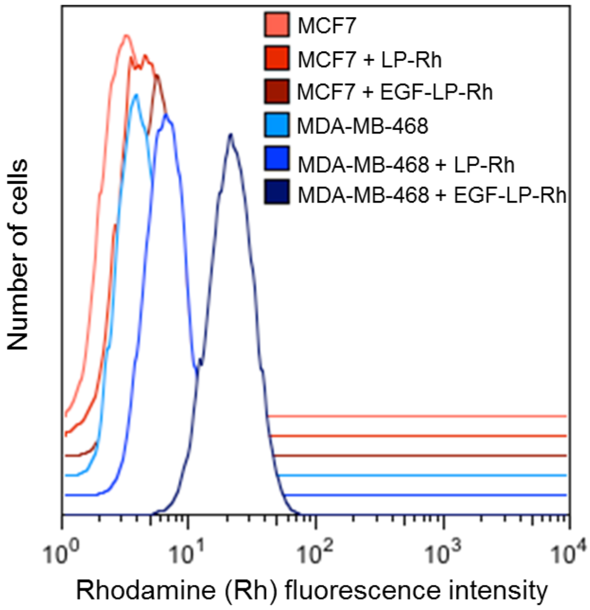

Figure S5: **Treatment of MDA-MB-468 (A) and MCF7 (B) with empty EGF-LP** for 2 hours followed by MTT assay 4 days after treatment. The data points represent cell viability following treatment, as a percentage of control (untreated cells). The formulation did not affect cell viability in the tested cell lines.

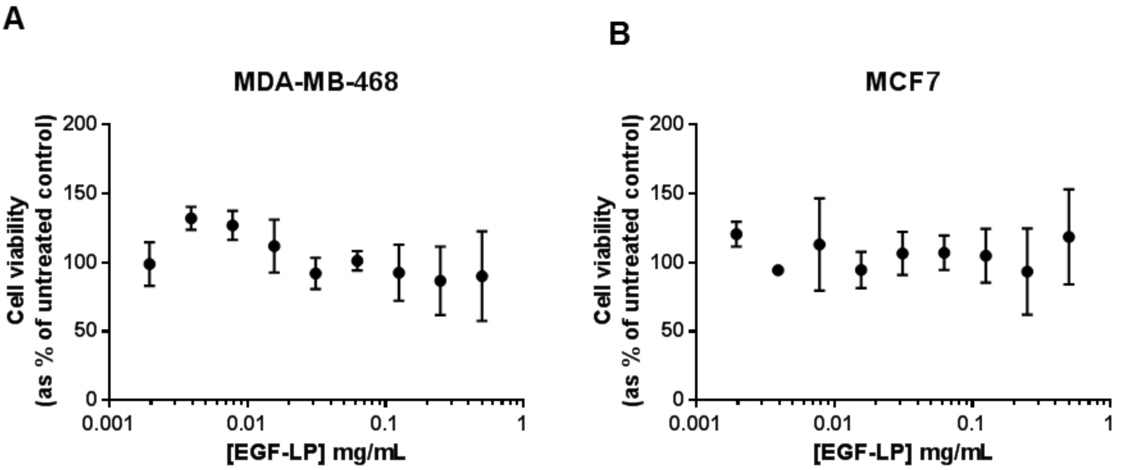

Figure S6: **Studies of the cytotoxicity of liposomes without doxorubicin**. Clonogenic studies on **(A)** MDA-MB-468 and **(B)** MCF7 cells following 24 hour treatment and 14 days incubation. (n=3, standard deviation shown). Same concentration in EGF was used for the groups of cells treated with EGF, EGF-LP or ^{111}In-EGF-LP.

A

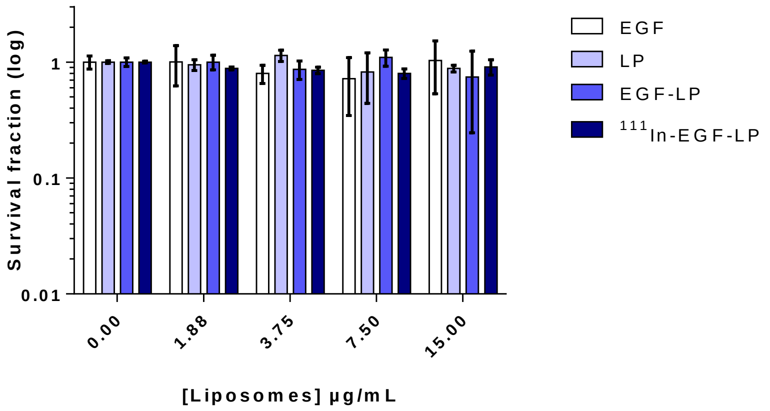

B

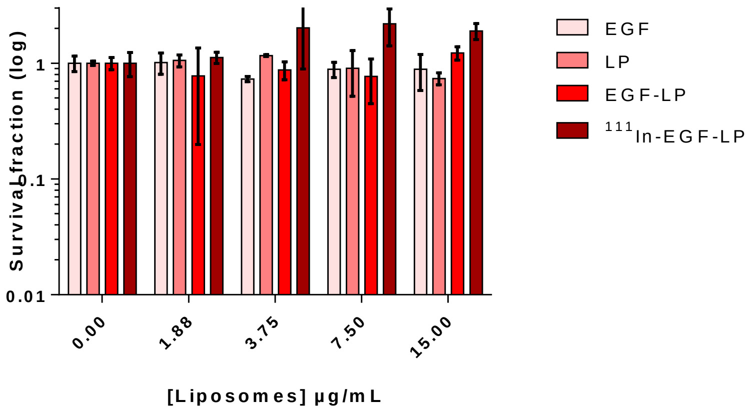

Figure S7: **DNA damage studies for MDA-MB-468 and MCF7 after treatment with liposomal formulation. (A)** Confocal microscopy images of MDA-MB-468 and MCF7 cells showing the presence of γH2AX foci (green) following treatment. **(B)** Number of γH2AX foci/cell counted using ImageJ software. Liposomal construct are capable of causing DNA damage in MDA-MB-468 and to a lesser extent in MCF7, with ^{111}In-EGF-LP-Dox having the largest effect. Incubation for 24 h at 37 °C (n = 3, standard deviation shown **** = p<0.00005, *** = p<0.0005, ** = p<0.005 and * = p<0.05 using ANOVA with Fisher's LSD analysis).

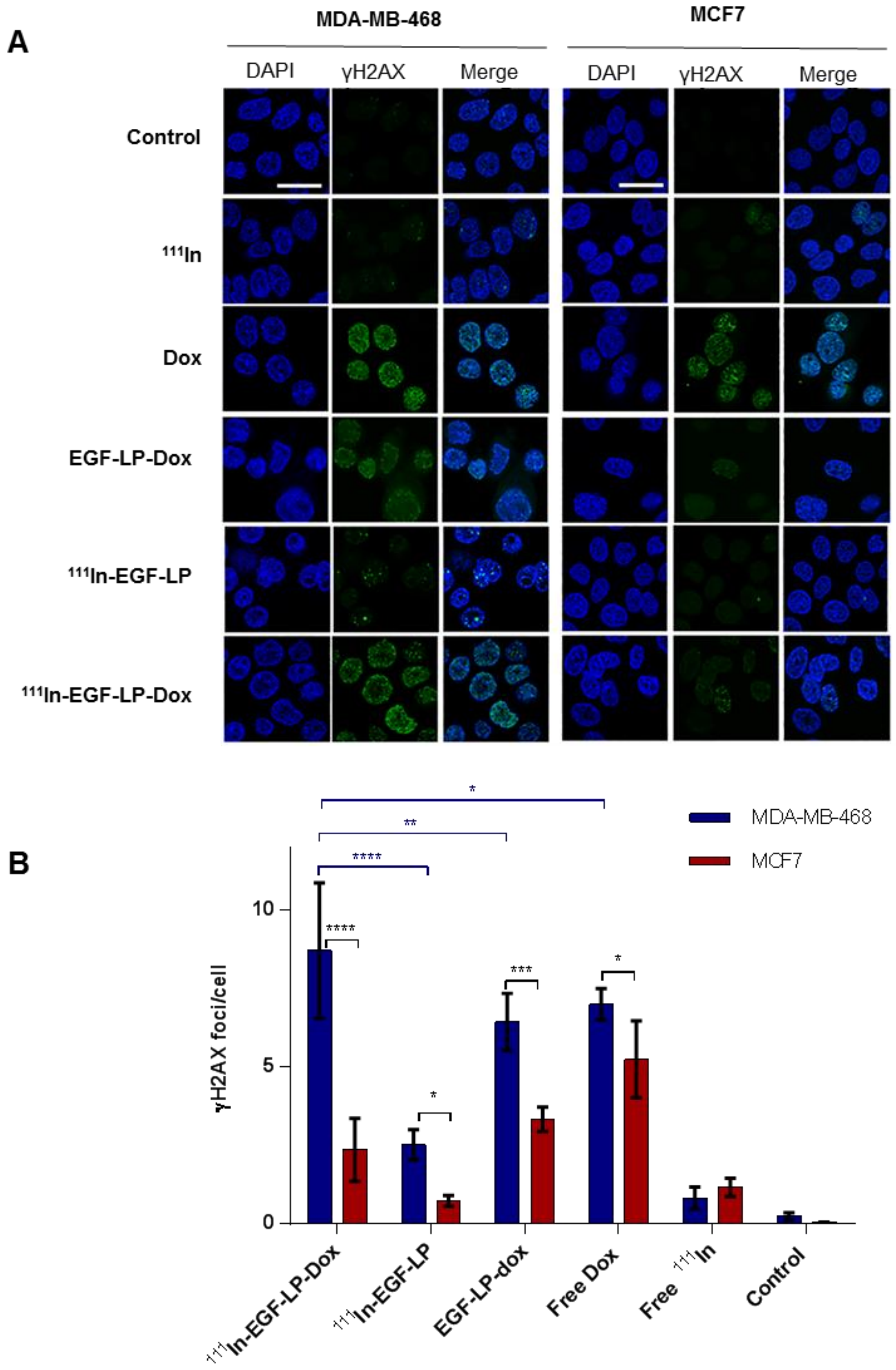

Figure S8: Schematic of the experimental setup used to study the cavitation-induced extravasation of DTPA-EGF and rhodamine-tagged liposomes in a gel vessel phantom (FUS: focused ultrasound; PCD: passive cavitation detector).

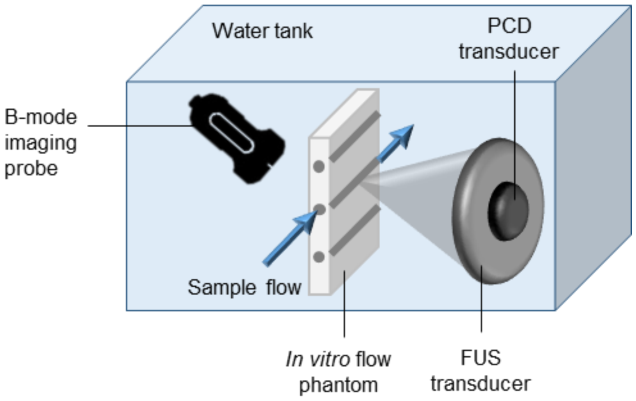

Figure S9: *In vivo* stability of ^{111}In-EGF-LP in mice bearing MDA-MB-468 xenografts. Mice were injected with the radiolabelled liposomes (5-8 MBq, 120 µg), serum was collected at different time points and analysed post-elution through a G75 column (elution with sodium citrate, 0.1 M, pH = 5, representative data shown). ^{111}In-EGF-LP is stable in the blood stream for at least 2 h 30 (black line) but free ^{111}In-EGF is detected after at 24 h and 48 h (grey and red lines). These degradation products are not expected to induce acute toxic effect [6, 7].

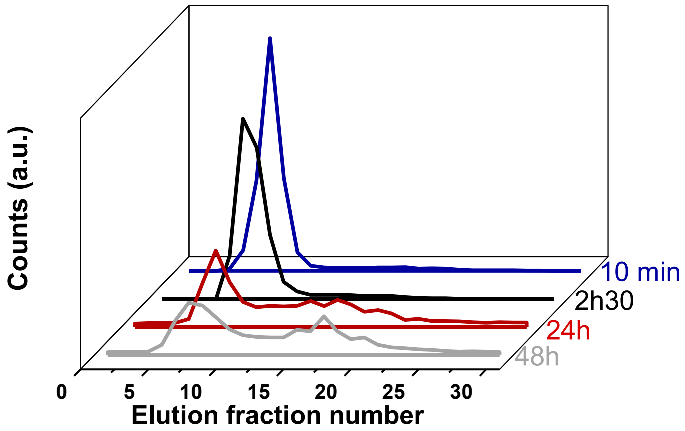

Figure S10: Biodistribution of ^{111}In-EGF-LP in MDA-MB-468 tumours after exposure to US in the presence of SV Autoradiography showing the distribution of ^{111}In-EGF-LP in one slice of the tumour 10 min post i.v. injection of the liposome (representative image).

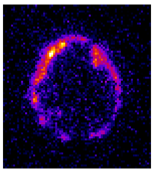

Table S1: Biodistribution of ^{111}In-EGF-LP in mice bearing MDA-MB-468 xenografts expressed as percentage of injected dose per organ (%ID/organ). The %ID in the blood is estimated based on the weight of the mice (n = 4-5, standard deviation shown). ^{111}In-EGF-LP are mainly eliminated through the liver.

	%ID/organ	
	2 h 30	48 h
Liver	63.58 ± 2.55	46.04 ± 2.17
Spleen	9.83 ± 3.35	3.30 ± 0.56
Kidneys	5.71 ± 0.47	4.21 ± 0.61
Blood	3.26 ± 0.65	0.16 ± 0.03
Small intestine	1.54 ± 0.15	0.63 ± 0.08
Large intestine	0.48 ± 0.04	0.75 ± 0.39
Lung	0.47 ± 0.09	0.10 ± 0.02
Pancreas	0.24 ± 0.03	0.17 ± 0.04
Stomach	0.19 ± 0.07	0.21 ± 0.13
Heart	0.13 ± 0.02	0.04 ± 0.01
Tumour	0.05 ± 0.02	0.06 ± 0.02
Brain	0.03 ± 0.01	0.01 ± 0.01
Total recovered	85.5	55.7

Table S2: Biodistribution of ^{111}In-EGF-LP in mice bearing MDA-MB-468 xenografts expressed as percentage of injected dose per gram of organ (%ID/g) (n = 4-5, standard deviation shown). Retention of ^{111}In-EGF-LP is higher in the tumour than in the muscle.

	%ID/g	
	2 h 30	48 h
Spleen	57.90 ± 7.85	35.02 ± 6.98
Liver	47.52 ± 10.67	32.71 ± 2.63
Kidneys	14.72 ± 1.62	10.60 ± 1.67
Lung	2.64 ± 0.26	0.61 ± 0.12
Small intestine	1.35 ± 0.21	0.64 ± 0.10
Blood	1.33 ± 0.15	0.07 ± 0.02
Bone	1.25 ± 0.41	0.03 ± 0.02
Pancreas	0.90 ± 0.12	0.68 ± 0.21
Heart	0.81 ± 0.06	0.30 ± 0.04
Fat	0.39 ± 0.06	0.08 ± 0.04
Large intestine	0.38 ± 0.07	0.74 ± 0.35
Tumour	0.36 ± 0.08	0.32 ± 0.06
Skin	0.35 ± 0.06	0.32 ± 0.05
Stomach	0.34 ± 0.06	0.52 ± 0.39
Muscle	0.12 ± 0.02	0.07 ± 0.01
Brain	0.06 ± 0.01	0.03 ± 0.02

References

1. Abraham SA, Waterhouse DN, Mayer LD, Cullis PR, Madden TD, Bally MB. The liposomal formulation of doxorubicin. Methods Enzymol 2005; 391: 71-97.
2. Niu G, Cogburn B, Hughes J. Preparation and characterization of doxorubicin liposomes. Methods Mol Biol. 2010; 624: 211-9.
3. Carugo D, Owen J, Crake C, Lee JY, Stride E. Biologically and acoustically compatible chamber for studying ultrasound-mediated delivery of therapeutic compounds. Ultrasound Med Biol. 2015; 41: 1927-37.
4. Vaissier PE, Beekman FJ, Goorden MC. Similarity-regulation of OS-EM for accelerated SPECT reconstruction. Phys Med Biol. 2016; 61: 4300-15.
5. Kinchesh P, Gilchrist S, Beech JS, Gomes AL, Kersemans V, Newman RG, et al. Prospective gating control for highly efficient cardio-respiratory synchronised short and constant TR MRI in the mouse. Magn Reson Imaging. 2018; 53: 20-7.
6. Vallis KA, Reilly RM, Scollard D, Merante P, Brade A, Velauthapillai S, et al. Phase I trial to evaluate the tumor and normal tissue uptake, radiation dosimetry and safety of (111)In-DTPA-human epidermal growth factor in patients with metastatic EGFR-positive breast cancer. Am J Nucl Med Mol Imaging. 2014; 4: 181-92.
7. Reilly RM, Chen P, Wang J, Scollard D, Cameron R, Vallis KA. Preclinical pharmacokinetic, biodistribution, toxicology, and dosimetry studies of 111In-DTPA-human epidermal growth factor: An auger electron–emitting radiotherapeutic agent for epidermal growth factor receptor–positive breast cancer. J Nucl Med. 2006; 47: 1023-31.
